# Quantitative imaging of vesicle–protein interactions reveals close cooperation among proteins

**DOI:** 10.1002/jev2.12322

**Published:** 2023-04-25

**Authors:** Minkwon Cha, Sang Hyeok Jeong, Jaehun Jung, Yoonjin Baeg, Sung‐Soo Park, Seoyoon Bae, Chan Seok Lim, Jun Hyuk Park, Jie‐Oh Lee, Yong Song Gho, Seung Wook Oh, Min Ju Shon

**Affiliations:** ^1^ Department of Physics Pohang University of Science and Technology (POSTECH) Pohang Republic of Korea; ^2^ POSTECH Biotech Center Pohang University of Science and Technology (POSTECH) Pohang Republic of Korea; ^3^ Biodrone Research Institute MDimune Inc. Seoul Republic of Korea; ^4^ Department of Life Sciences Pohang University of Science and Technology (POSTECH) Pohang Republic of Korea; ^5^ Institute of Membrane Proteins Pohang University of Science and Technology (POSTECH) Pohang Republic of Korea; ^6^ School of Interdisciplinary Bioscience and Bioengineering Pohang University of Science and Technology (POSTECH) Pohang Republic of Korea

**Keywords:** CD9, cell‐derived vesicles, ICAM‐1, integrin, total internal reflection fluorescence microscopy, vesicle–protein interactions

## Abstract

Membrane‐bound vesicles such as extracellular vesicles (EVs) can function as biochemical effectors on target cells. Docking of the vesicles onto recipient plasma membranes depends on their interaction with cell‐surface proteins, but a generalizable technique that can quantitatively observe these vesicle–protein interactions (VPIs) is lacking. Here, we describe a fluorescence microscopy that measures VPIs between single vesicles and cell‐surface proteins, either in a surface‐tethered or in a membrane‐embedded state. By employing cell‐derived vesicles (CDVs) and intercellular adhesion molecule‐1 (ICAM‐1) as a model system, we found that integrin‐driven VPIs exhibit distinct modes of affinity depending on vesicle origin. Controlling the surface density of proteins also revealed a strong support from a tetraspanin protein CD9, with a critical dependence on molecular proximity. An adsorption model accounting for multiple protein molecules was developed and captured the features of density‐dependent cooperativity. We expect that VPI imaging will be a useful tool to dissect the molecular mechanisms of vesicle adhesion and uptake, and to guide the development of therapeutic vesicles.

## 1 INTRODUCTION

Membrane‐bound vesicles can effectively elicit biochemical changes in cells. For example, extracellular vesicles (EVs), which are naturally secreted by virtually all types of cells, can adhere to the plasma membranes of target cells and trigger intracellular signalling, often accompanied by cargo transfer upon internalization. This process is therefore believed to enable effective remote communication among distant cells (van Niel et al., 2018). Such properties have also inspired the development of vesicles with therapeutic potentials (Murphy et al., 2019). For both types of vesicles involved in physiological communication and pharmacological intervention, their biochemical effects are mediated by a group of proteins at the interface of vesicles and plasma membranes. The collective affinity among the participating molecules manifests as overall vesicle–protein interactions (VPIs) and ultimately determines the fate of the docked vesicles.

Although VPIs may be dominated by strongly interacting proteins, an underlying variability among vesicles and target proteins can potentially diversify the outcome. For example, many cell adhesion molecules can form oligomers that organize into nanoscale clusters (Garcia‐Parajo et al., 2014), which can then serve as docking platforms for vesicles. Moreover, regulatory proteins near major interactors can alter the stability of VPIs, thus leading to differential efficiencies of vesicle uptake (Mulcahy et al., 2014). Nano‐sized vesicles display only a small, random number of protein molecules on their surfaces, and are therefore intrinsically heterogeneous in their physicochemical properties (Willms et al., 2018). Consequently, to characterize the functional heterogeneity across vesicles and target proteins, a technique that can precisely measure VPIs at the single‐vesicle level is desirable.

Standard methods for characterizing vesicles include high‐resolution imaging and tracking of vesicles to measure particle size (e.g. electron microscopy, dynamic light scattering (DLS), and nanoparticle tracking analysis (NTA)), biochemical assays for profiling composition (e.g. immunostaining and western blot), and cell‐based experiments to measure functional properties of vesicles (e.g. cargo delivery, changes in cell signalling) (Shao et al., 2018; Théry et al., 2018). However, a protocol that addresses VPIs, which relate these properties, has not been introduced yet. We here aimed to fill this gap by developing an in vitro assay that quantitatively reports on VPIs between individual vesicles and proteins. This technique can not only complement the existing methods of vesicle characterization but also provide functionally relevant results much faster at a much‐reduced cost compared with live‐cell assays. Furthermore, by applying a reductionist approach in well‐controlled test tubes, one can decompose the contributions from the involved components (proteins, lipid membranes, and buffer constituents), which will help reveal molecular mechanisms of vesicle adhesion and internalization.

Here, we present a method for quantitative fluorescence imaging of VPIs. Fluorescently labelled vesicles are introduced to target proteins prepared on a glass coverslip, and their interactions are measured using total internal reflection fluorescence (TIRF) microscopy. We validated our method by probing the interactions between cell‐derived vesicles (CDVs) and intercellular adhesion molecule‐1 (ICAM‐1), a cell‐surface protein that mediates cell–cell adhesion (Rothlein et al., 1986) and EV uptake (Mulcahy et al., 2014). VPIs were probed with ICAM‐1 molecules that were either tethered to polymer‐coated glass surface, or embedded in supported lipid bilayers. The observed VPIs first recapitulated the known factors of vesicle adhesion, such as the participation of an integrin, lymphocyte function‐associated antigen 1 (LFA‐1; αLβ2; CD11a/CD18), a key receptor for ICAM‐1. Unexpectedly, however, the strength of VPIs measured for vesicles of different origins did not strictly correlate with their LFA‐1 abundance. We also discovered that CD9, a tetraspanin protein with a broad tissue distribution, strongly promoted VPIs with ICAM‐1 particularly when ICAM‐1 and CD9 were closely located and embedded in lipid membranes. Methods described in this work provide opportunities to elucidate molecular determinants of vesicle functionality in a much simpler way than laborious assays that use cells.

## 2 RESULTS

### 2.1 Design of vesicle–protein interaction (VPI) imaging

**FIGURE 1:**
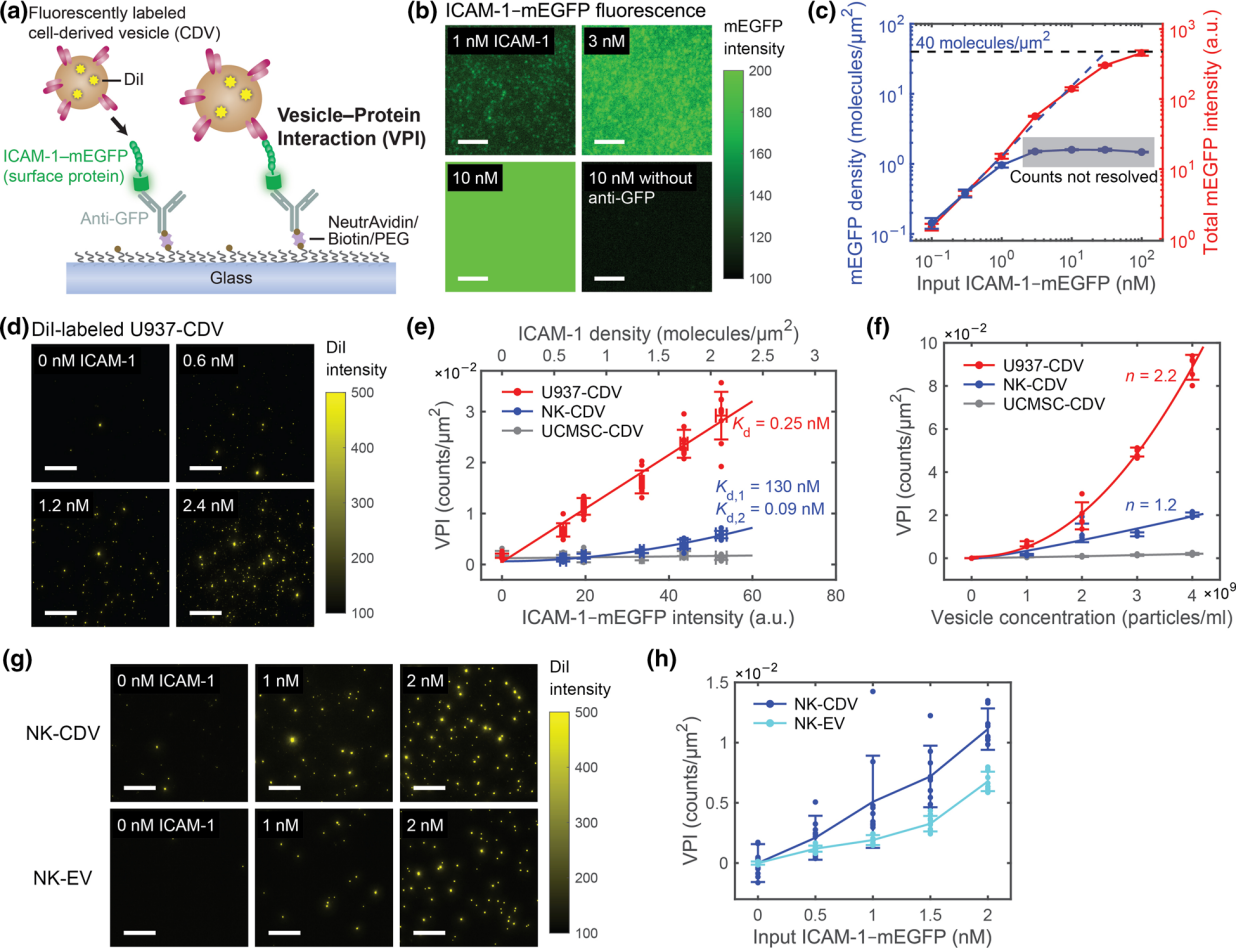
VPIs between CDVs and surface‐tethered ICAM‐1 (a) Schematic of VPIs between DiI‐labelled CDVs and ICAM‐1–mEGFP tethered to PEG‐coated glass surface. (b) Fluorescence images of surface‐tethered ICAM‐1–mEGFP prepared from the indicated concentrations of ICAM‐1–mEGFP. Scale, 5 μm. (c) Calibration of ICAM‐1–mEGFP density. Single‐molecule counts of GFP spots (*blue, left axis*) were compared with total fluorescence intensity in the same regions (*red, right axis*) as a function ICAM‐1–mEGFP concentration. Dashed, density estimation from intensity. Gray box indicates a regime where single spots cannot be optically resolved. (d) Fluorescence images of DiI‐labelled U937‐CDVs on ICAM‐1–mEGFP surface prepared from the indicated concentrations. Scale, 20 μm. (e) VPIs of various CDVs as a function of ICAM‐1 amount. The upper axis was calculated from the intensity in the lower axis. U937‐ and NK‐CDV data were fitted (*solid*) with a simple adsorption and a density‐dependent cooperation model, respectively, to obtain the equilibrium constants. (f) VPIs as a function of vesicle concentration. Data were fitted (*solid*) with a cooperative vesicle binding model to obtain the Hill coefficient (*n*). (g) Fluorescence images of DiI‐labelled NK‐CDVs and NK‐EVs on ICAM‐1–mEGFP surface. Scale, 20 μm. (h) VPI counts of NK‐CDVs and NK‐EVs as a function of input amount of ICAM‐1. In all panels, error bars represent mean ± s.d. for *n* = 5−10 images, which are technical replicates acquired from distinct surface locations prepared with the same materials.

We first devised a scheme to measure VPIs (Figure 1a). In this procedure, target protein molecules are tethered to a glass surface by antibodies, and then fluorescently labelled vesicles are added to allow them to interact with the surface‐tethered proteins. To validate the method, we first employed cell‐derived vesicles (CDVs) as model vesicles (Jang et al., 2013), and then tested a sample of natural EVs. CDVs are EV‐mimetic nano‐vesicles produced by extruding source cells through small pores. Since they inherit different sets of membrane proteins and lipids from parental cells (Lee et al., 2020), a group of CDVs were prepared to test the general applicability of our method. For proof‐of‐principle experiments, we measured their interactions with ICAM‐1, a major cell adhesion molecule that controls adhesion between leukocytes and endothelial cells. In the context of vesicles, ICAM‐1 can serve as a target for immune‐cell‐derived EVs or therapeutic vesicles (Morelli et al., 2004; Rana et al., 2012; Taftaf et al., 2021). Finally, a TIRF microscope was used to selectively illuminate surface‐tethered proteins and the interacting vesicles, rejecting background fluorescence from free‐floating particles. If the VPIs are stable, one can also measure the vesicle count after washing away unbound vesicles.

### 2.2 Preparation of surface‐tethered ICAM‐1

To prepare ICAM‐1‐coated surfaces, recombinant ICAM‐1 fused with monomeric enhanced green fluorescent protein (ICAM1–mEGFP) was expressed from HEK293 (HEK) cells. The cells were gently lysed with non‐ionic detergent (1% Triton X‐100), and the solubilized ICAM1–mEGFP molecules were pulled down onto polyethylene glycol (PEG)‐coated glass by using a biotinylated antibody to green fluorescent protein (GFP) (Figure 1a). The use of GFP enables simple and unbiased capture of virtually any target proteins without laborious purification, and also allows use of fluorescence to quantify the pulled‐down molecules. The mEGFP tag was fused to the C‐terminus of ICAM‐1 and therefore neither perturbed the membrane localization of ICAM‐1 nor interfered with its extracellular domain (Figure S1A, B).

At low density, the ICAM‐1 surface displayed well‐resolved GFP spots (Figure 1b). Photobleaching experiments to measure the numbers of fluorophores per puncta (i.e. the number of GFP molecules per spot) (Mutch et al., 2007; Ulbrich & Isacoff, 2007; Kim et al.,^2021^) revealed that the initial fluorescent intensities were comparable to the bleaching step size (Figure S1C, D; see “Single‐molecule photobleaching analysis” in Supporting Information), indicating that these spots indeed represent individual ICAM‐1 molecules. The ICAM‐1 density increased linearly with the input amount of ICAM‐1 (Figure 1b,c). By directly counting the number of spots, we accurately measured the density up to ∼1 molecules/μm^2^, but the spots were not resolved beyond that because of the diffraction limit (Figure 1c, *blue*). In contrast, the overall brightness of the field (i.e. total fluorescence intensity) kept increasing with more ICAM‐1, until it was limited by the surface occupancy (Figure 1c, *red*). To estimate the ICAM‐1 density in dense samples, we therefore calibrated the total intensity as a function of spot density in the low‐density regime (Figure S1E), and extrapolated the linearity to the high‐density regime (Figure 1c, *dashed*). This scheme is reliable because the GFP fluorescence did not exhibit self‐quenching behaviour at high densities (Figure S1F), indicating a robust linear relationship between ICAM‐1 density and the resulting fluorescence. In this way, the maximum density of ICAM‐1 that we can obtain was estimated to be ∼40 molecules/μm^2^ (Figure 1c). At high density, some neighbouring ICAM‐1 molecules may cooperate to capture a vesicle, if the nearest neighbour is located with the reach of a captured vesicle by chance (see “Modelling of VPI” in Supporting Information). Also, a fraction of ICAM‐1 might have been pulled down as dimers or larger oligomers (Miller et al., 1995; Reilly et al., 1995). Indeed, when ICAM‐1 surface was prepared from a concentrated (∼30 nM) solution, the resulting spots were at least twice as bright on average compared with the ones from a dilute (0.3 nM) sample (Figure S1G), suggesting that multimeric ICAM‐1 may indeed exist. In contrast, the detergent‐solubilized ICAM‐1–mEGFP was verified by immunoprecipitation experiments not to precipitate with other endogenous proteins (Figure S1H), and therefore the ICAM‐1 surface was also considered to be free of associated factors other than ICAM‐1.

### 2.3 VPIs with surface‐tethered ICAM‐1

VPI imaging may be useful to compare target‐binding properties of various samples of vesicles, so we prepared three types of CDVs in parallel by using a published procedure (Zhang et al., 2021): one from human natural killer cells (NK‐CDV), another from pro‐monocytic U937 cells (U937‐CDV), and the other from umbilical cord mesenchymal stem cells (UCMSC‐CDV). NTA results showed nearly identical size distributions for the three vesicles (Figure S2A): median diameters of 140 nm (NK‐CDV), 160 nm (U937‐CDV), and 124 nm (UCMSC‐CDV), similar to natural EVs (van Niel et al., 2018). The protein compositions of the CDVs largely resembled the proteomes of the respective parental cells (Figure S2B), suggesting that the production of CDVs was unbiased both physically and chemically (Nasiri Kenari et al., 2019). Transmission electron microscopy (TEM) and cryo‐electron microscopy (cryo‐EM) followed by deep‐learning‐based image analysis (Gómez‐de‐Mariscal et al., 2019) and manual inspection of lamellar structures further confirmed the spherical shapes of the vesicles (Figure S2C–F and Table S1; see also “Particle analysis of vesicle samples” and “Chemical analysis of vesicle samples” in Supporting Information for more details).

The three CDVs (NK‐, U937‐, and UCMSC‐CDVs) were next labelled with DiI (DiIC_18_(3)), a lipophilic fluorescent dye that stains lipid membranes (Video S1) (Cha et al., 2023). Finally, the labelled CDVs were introduced to the surface preparations of ICAM‐1. During incubation, TIRF imaging visualized transient vesicles diffusing in the background, as well as immobile ones on surface. To focus on analysing stable binders, we washed away the vesicle solution after a specific period. The interacting vesicles appeared as bright spots that were well separated from one other, so we counted the number of bound vesicles and used it as a measure of VPI strength (Video S2 shows the overall measurement procedure). For example, U937‐CDVs accumulated when ICAM‐1 concentration was increased (Figure 1d), indicating a strong, specific interaction with ICAM‐1. Although some spots were brighter than the others, most of them still appeared as diffraction‐limited spots with a 2‐D Gaussian profile (Figure S3A), so all of the non‐overlapping spots were successfully detected by an automated software. In fact, the distribution of spot intensity showed a strong peak (Figure S3B), indicating that most of the vesicles were of similar brightness. Since a spot was registered as one VPI count if and only if its brightness was above a threshold, this scheme is robust to the heterogeneity in vesicle labelling, background fluorescence, and weakly fluorescent contaminants. Vesicle binding reached equilibrium within 15 min (Figure S3C), and most of the bound vesicles remained stable even for 1 d under gentle flow (1 μl/min) (Figure S3D–F). This rapid and stable association suggests that the observed VPIs may be potent in triggering biochemical changes of cells.

### 2.4 Cooperativity in VPIs

Among the three CDV samples, U937‐ and NK‐CDVs consistently exhibited strong interactions with ICAM‐1, whereas UCMSC‐CDVs did not show any attachment within the concentration range that we explored (Figure 1e). The VPI levels of U937‐ and NK‐CDVs depended on ICAM‐1 density, as expected. For example, binding of U937‐CDVs was detected even on a sparse ICAM‐1 surface with ∼0.5 molecules/μm^2^, whereas NK‐CDV binding became discernible in the density regime of >1.0 molecules/μm^2^. Note that the overall level of vesicle binding was low compared with ICAM‐1 density, because we used a very low “molar” concentration of vesicles, ∼3 pM (∼2×10^9^ particles/ml). Accordingly, only a small fraction of ICAM‐1 would be bound to vesicles. In this case, the use of high‐density surface protein can compensate for the limited sample concentration and promote the interaction to a quantifiable level, especially when the interaction is weak (Yoo et al., 2016). Furthermore, this design allowed the accurate counting of well‐separated vesicle spots, as well as probing of molecular cooperativity at high ICAM‐1 density (see below).

Intriguingly, while the binding of U937‐CDVs increased linearly with ICAM‐1 density, VPIs of NK‐CDVs exhibited a faster increase at a higher density (Figure 1e, *blue*). These results imply that adjacent ICAM‐1 molecules might cooperate in NK‐CDV binding, whereas isolated ICAM‐1 monomers might be sufficient to capture U937‐CDVs (Jun et al., 2001). For quantitative descriptions of VPIs, we developed a density‐dependent adsorption model in which two or more protein molecules can cooperate to bind single vesicles (Figure S4; see “Modelling of VPI” in Supporting Information). The model accounts for protein density, binding geometry, and the equilibrium among the unbound and bound vesicles. Indeed, the super‐linear increase of NK‐CDV binding could be explained only by a density‐dependent model featuring at least two equilibrium constants (*K*_d,1_ = 130 nM and *K*_d,2_ = 0.09 nM), whereas a simple Langmuir adsorption (with *K_d_* = 0.25 nM) was sufficient to describe the VPIs of U937‐CDVs (Figure 1e) (investigation of this difference is presented in the section below: “Activated LFA‐1 in U937‐CDVs drives strong VPIs”). The equilibrium constants obtained here for VPIs were formulated with vesicle concentration and surface‐protein density, so they must not be regarded as bimolecular equilibrium constants for the participating proteins.

For the same concentrations of ICAM‐1, increasing the input concentration of vesicles led to stronger VPI signals, as expected (Figure 1f). VPIs with NK‐ and U937‐CDVs showed a super‐linear increase in the concentration regime that we studied (up to 4 × 10^9^ particles/ml). When fitted with a Hill equation, both U937‐ and NK‐CDV binding indicated a weak cooperativity (with Hill coefficients *n* = 2.2 and 1.2, respectively; see “Modelling of vesicle–protein interactions” in Supporting Information). We speculate that such behaviour may be explained by transient association of vesicles that overall recruits more vesicles toward the ICAM‐1 surface, as suggested for the surface adsorption of proteins (Rabe et al., 2011).

### 2.5 VPI measurements on natural EVs

To test whether VPI imaging can be applied to naturally occurring vesicles, we collected EVs secreted by NK‐92 cells (referred to as NK‐EV), following the MISEV guideline (Théry et al., 2018). NK‐EVs had a similar size to CDVs (median diameter of 157 nm; Figure S5A), were largely spherical as revealed by TEM (Figure S5B), and were enriched with EV markers (Figure S5C). We stained NK‐EVs with fluorescent dyes in the same way as CDVs, and their interaction with ICAM‐1‐coated surface was compared side‐by‐side with NK‐CDVs. The labelled NK‐EVs were largely indistinguishable from NK‐CDVs (Figure 1g), and their VPI counts also increased with the input amount of ICAM‐1. Interestingly, the ICAM‐1 dependence of NK‐EVs were comparable to that of NK‐CDVs (Figure 1h) despite their distinct production process, which warrants future attention.

### 2.6 ICAM‐1‐mediated VPIs depend on LFA‐1

We next sought to identify the CDV proteins that mediate ICAM‐1‐dependent VPIs. ICAM‐1 was originally identified as a potent ligand of LFA‐1, a heterodimeric complex consisting of CD11a (αL integrin) and CD18 (β2 integrin). LFA‐1 is not only expressed in all major types of leukocytes (Marlin & Springer, 1987; Walling & Kim, 2018), but also its interaction with ICAM‐1 on EV surface has been frequently implicated in immunological contexts (Lee et al., 2010, p. 1; Nolte‐‘t Hoen et al., 2009; Segura et al., 2007; Zhang et al., 2022). Since both NK‐ and U937‐CDVs were derived from blood cells, we accordingly hypothesized that LFA‐1 molecules in these vesicles recognize surface‐tethered ICAM‐1 and drive the observed VPIs.

**FIGURE 2:**
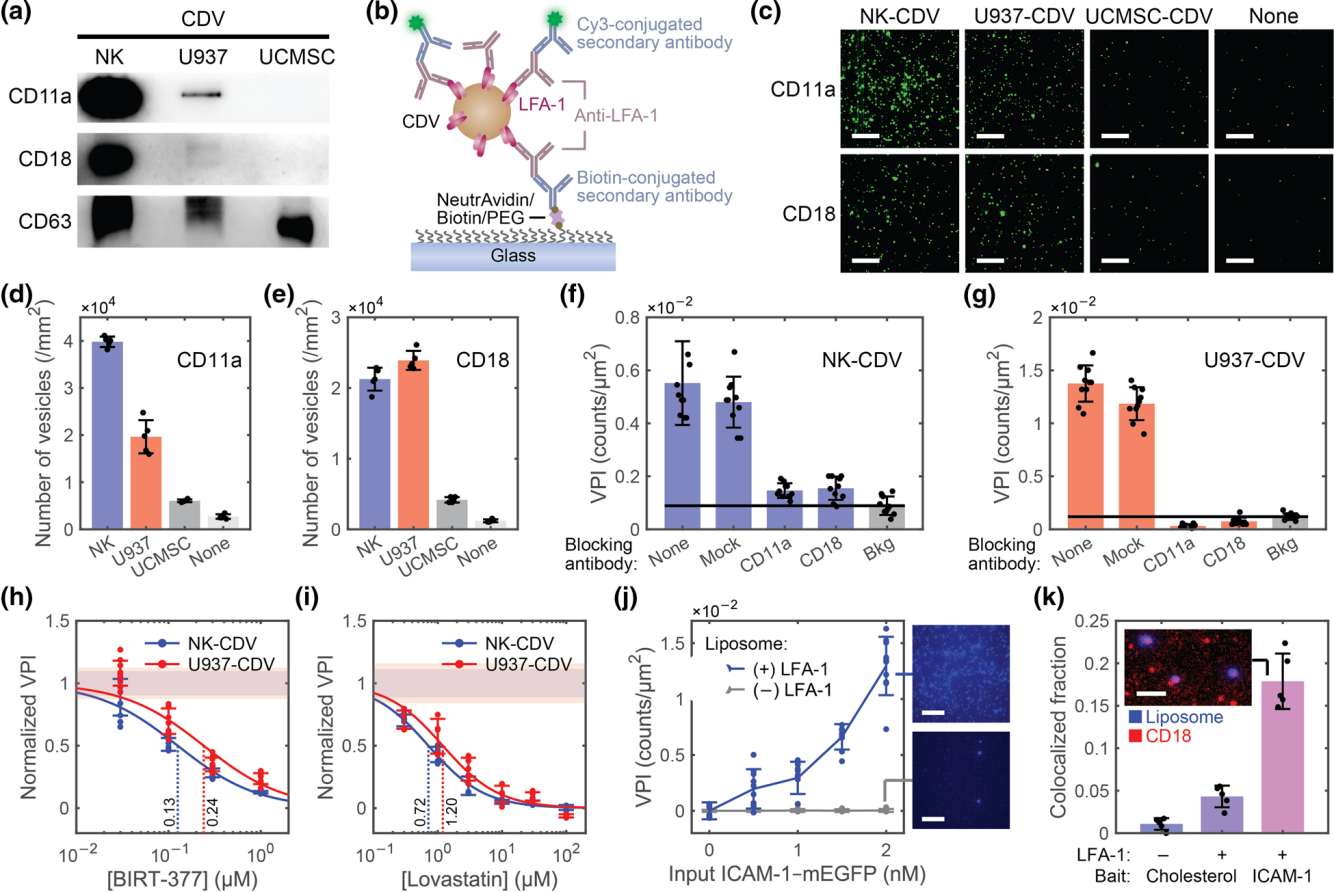
ICAM‐1‐mediated VPIs depend on LFA‐1 (a) Western blot analysis of CDVs for LFA‐1 subunits: CD11a (αL) and CD18 (β2). An antibody to CD63 was used as a positive control. (b) Schematic of single‐vesicle immunoblotting for the detection of LFA‐1. (c) Representative images from single‐vesicle immunoblotting. Scale, 10 μm. (d, e) Counts of CD11a‐ and CD18‐positive vesicles obtained from images such as shown in (c). (f, g) VPIs measured for NK‐ and U937‐CDVs after incubation with the indicated blocking antibodies. (h, i) VPIs measured in the presence of LFA‐1 antagonists BIRT‐377 and lovastatin. Fits are sigmoidal models with the fitted IC_50_ values (*dotted*). (j) VPIs between LFA‐1‐loaded liposomes and ICAM‐1 surface. Control liposomes were prepared the same way but without LFA‐1. Insets, representative images of DiD‐labelled liposomes at the highest ICAM‐1 density. (k) Fraction of liposomes colocalized with CD18−mCherry fluorescence. For comparison, the same liposomes that were nonspecifically captured by surface‐tethered cholesterol were analysed. Inset, an image of ICAM‐1‐pulled, LFA‐1‐positive liposomes with high colocalization (scale bar, 5 μm). In all panels, error bars represent mean ± s.d. for *n* = 5−10 images, which are technical replicates acquired from distinct surface locations prepared with the same materials.

To thoroughly test the involvement of LFA‐1 in the measured VPIs, we adopted five independent approaches (Figure 2). First, traditional western blot showed that only NK‐ and U937‐CDVs contained measurable amounts of the LFA‐1 subunits, CD11a and CD18 (Figure 2a). Unexpectedly, U937‐CDVs displayed less LFA‐1 than NK‐CDVs, seemingly in contrast to their superior VPIs with ICAM‐1 (Figure 1e,f). Secondly, we also employed single‐vesicle immunoblotting assay to detect intact LFA‐1 molecules on vesicle membranes (Figure 2b) (Han et al., 2021). This method uses the same surface chemistry (PEGylated glass with biotin–NeutrAvidin coupling) and optical setup as VPI imaging, and therefore can robustly quantify vesicular proteins that might participate in VPIs. Again, we confirmed the presence of LFA‐1 in both NK‐ and U937‐CDVs (Figure 2c−e). For the third and the fourth tests, we hypothesized that inhibiting LFA‐1 activity would impair the observed VPIs. We first used monoclonal antibodies that target the extracellular domains of CD11a and CD18, and they indeed markedly blocked vesicle binding (Figure 2f, g). In particular, VPIs of U937‐CDVs were completely abolished despite the limited amount of LFA‐1 (Figure 2a), implying a critical dependence on LFA‐1. In contrast, a control, unrelated antibody (rabbit polyclonal to RFP) showed no effect. Although this approach is simple and informing, recruitment of many anti‐LFA‐1 molecules on LFA‐1‐enriched membranes can also spatially (rather than chemically) interfere with VPIs driven by other proteins. To rule out this possibility, we next tested two small‐molecule LFA‐1 antagonists, BIRT‐377 and lovastatin (Arkin & Wells, 2004). When either of the drugs was added to the assay buffer, the resulting VPIs again decreased in a dose‐dependent manner for both NK‐ and U937‐CDVs (Figure 2h, i). Importantly, the measured half‐maximal inhibitory concentrations (IC_50_) agreed well with the values reported for the in vitro interaction of LFA‐1/ICAM‐1 (Kallen et al., 1999; Kelly et al., 1999).

Finally, as a rudimentary validation of in vitro VPI measurements, we prepared synthetic liposomes that are loaded only with purified LFA‐1 (Supporting Information), and tested their reactivity toward surface‐tethered ICAM‐1. The two subunits of LFA‐1 were constructed as recombinant fusion proteins (CD11a−mEGFP and CD18−mCherry), co‐expressed from HEK cells, and purified by 6xHis‐tag on CD11a. Both subunits were successfully detected after purification (Figure S6A) and showed good association both before and after their incorporation into liposomes (Figure S6B−F). The function of recombinant LFA‐1 was further checked by Mn^2+^‐induced conformational shift (Figure S6G, H) (Dransfield et al., 1992). Then, we labelled LFA‐1 liposomes with DiD and added them to ICAM‐1 surface similarly to CDV experiments. LFA‐1 liposomes indeed adhered to the ICAM‐1‐coated surface in a density‐dependent manner, but clearly the control liposomes without LFA‐1 did not (Figure 2j). Furthermore, although the initial occupancy of liposomes by LFA‐1 was only moderate because of the limited amounts of purified proteins, LFA‐1‐bearing liposomes became enriched when ICAM‐1 was used as a “bait” (Figure 2k). When compared with nonspecifically captured liposomes pulled down with surface‐anchored cholesterol, the fraction of DiD/mCherry colocalized spots increased 4.1 times. This result again suggests that LFA‐1 molecules specifically mediate VPIs with surface‐tethered ICAM‐1. Together, all of the above results point to the important role of LFA‐1 in establishing VPIs between NK‐ and U937‐CDVs and ICAM‐1.

### 2.7 Activated LFA‐1 in U937‐CDVs drives strong VPIs

**FIGURE 3:**
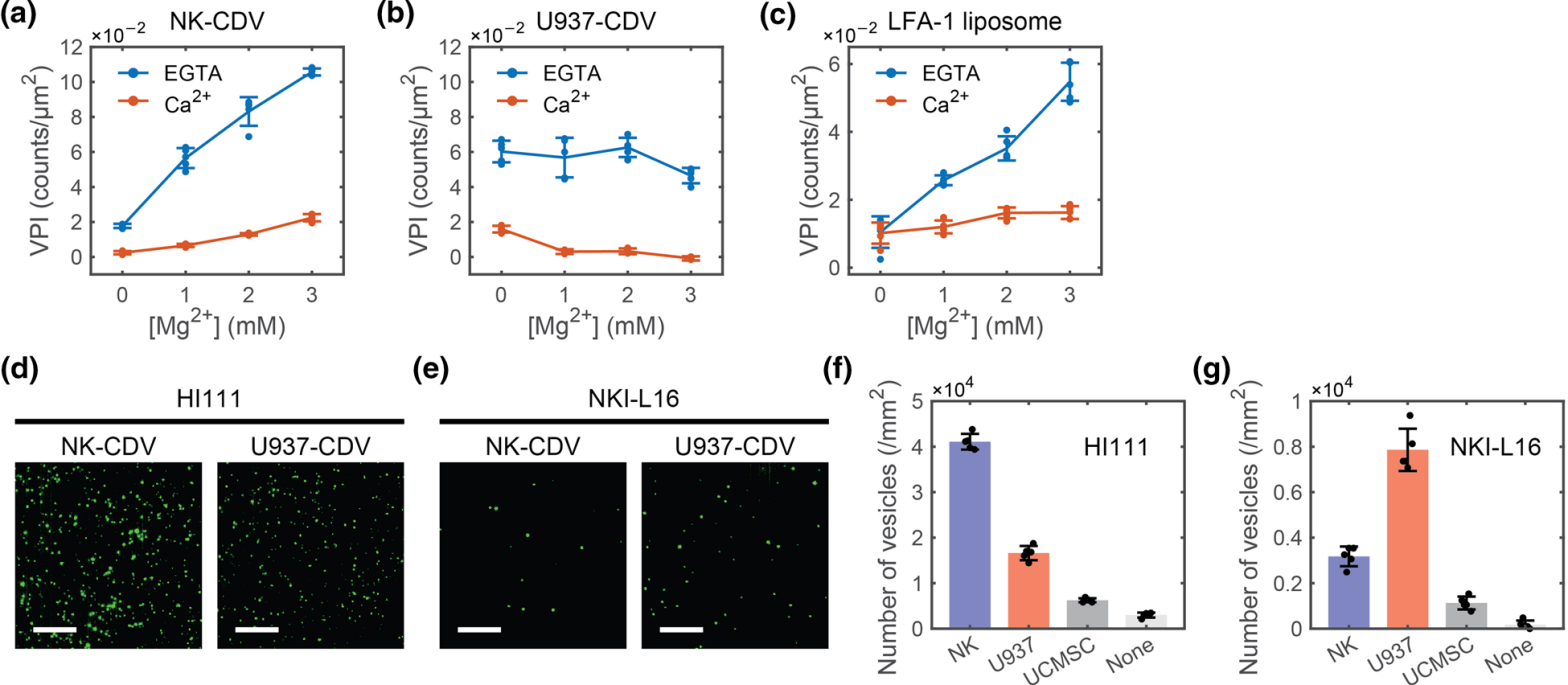
Activated LFA‐1 in U937‐CDVs drives strong VPIs (a–c) VPIs measured for NK‐CDVs, U937‐CDVs, and LFA‐1 liposomes as a function of Mg^2+^ and Ca^2+^ concentrations. Buffers were supplied either with 0.5 mM EGTA (*blue*) or with 0.2 mM Ca^2+^ (*red*). (d, e) Representative images from single‐vesicle immunoblotting with activation‐sensitive LFA‐1 antibodies: HI111 for the closed form (D); NKI‐L16 for the activated form (e). Scale, 10 μm. (f, g) Counts of HI111‐ and NKI‐L16‐positive vesicles obtained from images such as shown in (d) and (e). In all panels, error bars represent mean ± s.d. for *n* = 5−10 images, which are technical replicates acquired from distinct surface locations prepared with the same materials.

Although the VPIs of NK‐ and U937‐CDVs both depended on LFA‐1, the small amount of LFA‐1 found in U937‐CDVs (Figure 2a) appeared to contradict their strong VPI signals (Figure 1e, f). We therefore searched for potential differences between NK‐ and U937‐CDVs in their binding to ICAM‐1. We first checked whether the observed VPIs would respond to the regulation by divalent cations. It is well established that Mg^2+^ and Ca^2+^ orchestrate a conformational shift in integrins and thus regulate their binding to ICAM‐1 (Zhang & Chen, 2012). Specifically, Mg^2+^ directly coordinates ICAM‐1 on the binding site of LFA‐1 (Dransfield et al., 1992), whereas multiple Ca^2+^‐binding sites in LFA‐1 further modulate this interaction (Chen et al., 2003). When Mg^2+^ was added (1−3 mM) in the absence of Ca^2+^, VPIs of NK‐CDVs gradually increased until they surpassed those of U937‐CDVs without Mg^2+^ (Figure 3a, *blue*). We presume that this increase in VPIs resulted from conformational changes in LFA‐1, similar to its physiological behaviour (Shimaoka et al., 2003). In stark contrast, the strong VPIs observed with U937‐CDVs did not further increase upon addition of Mg^2+^ (Figure 3b, *blue*). This level (∼6×10^−2^ vesicles/μm^2^) was not limited by the detection range, because the overall density was sparse and NK‐CDVs were detected up to at least ∼10^−1^ vesicles/μm^2^ in the corresponding measurements. In contrast, 0.2 mM Ca^2+^ overall suppressed VPIs in both types of vesicles (Figure 3a and b, *red*), consistent with the physiological inhibition of integrin‐mediated adhesion by high concentrations of Ca^2+^. For comparison, we also tested in the same assay the response of LFA‐1 liposomes (Figure 3c), which were not expected to have been activated during the preparation. Indeed, similarly to NK‐CDVs, the binding of liposomes to ICAM‐1 was increased when Mg^2+^ was added, consistent with the basal state without Mg^2+^.

Based on the above results, we hypothesized that LFA‐1 in U937‐CDVs might exist predominantly in the activated form, rendering these vesicles insensitive to further stimulation by Mg^2+^. To determine whether this was the case, we carried out single‐vesicle immunoblotting again (Figure 3d–g), this time using two activation‐sensitive monoclonal antibodies: HI111 that preferentially targets the closed, low‐affinity state of LFA‐1 (Ma et al., 2002), and NKI‐L16 that recognizes the active, extended conformation of LFA‐1 (van Kooyk et al., 1991). Remarkably, NK‐CDVs were preferentially detected by HI111 (therefore suggesting low‐affinity LFA‐1) (Figure 3d, f), whereas U937‐CDVs were stained better with NKI‐L16 (therefore suggesting activated LFA‐1), exceeding the counts of NK‐CDVs (Figure 3e, g). These results suggest that activated LFA‐1 molecules might be enriched in U937‐CDVs and enabled the strong interaction with ICAM‐1. The mechanism that induces and sustains LFA‐1 activation in U937‐CDVs remains unclear (see Discussion).

### 2.8 CD9 strongly promotes ICAM‐1‐mediated VPIs

VPI experiments so far have measured interactions between given pairs of vesicles and target proteins. However, target binding of vesicles in a physiological milieu might also be influenced by local environmental factors such as chemokines or nearby proteins. These regulatory effects can be tested in our assay by exchanging buffers (as shown for Mg^2+^ and Ca^2+^ above) or by preparing surfaces with different protein compositions. In fact, we already witnessed that increasing ICAM‐1 density leads to nonlinear enhancement of VPIs (Figure 1e). This effect reminded us the function of a tetraspanin protein, CD9, that forms microdomains and thereby induces clustering of LFA‐1 and ICAM‐1 (Barreiro et al., 2005; Reyes et al., 2018). Since CD9 is expressed in most types of leukocytes and also in endothelial cells, we accordingly asked whether CD9 molecules near surface‐tethered ICAM‐1 might assist the capturing of blood‐cell‐derived NK‐CDVs, and in this way reproduce the endogenous function of CD9 in cell adhesion.

**FIGURE 4:**
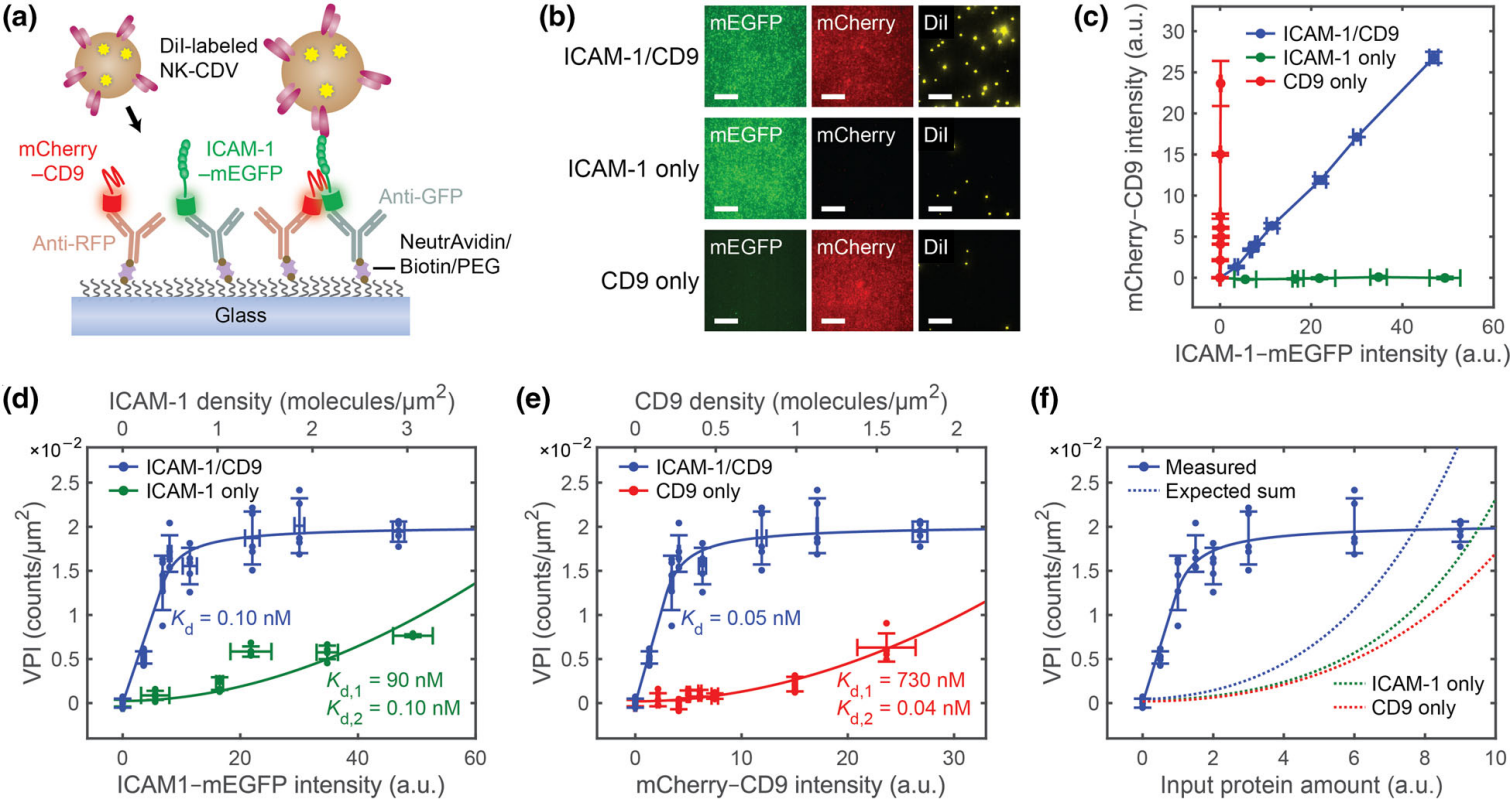
CD9 strongly promotes ICAM‐1‐mediated VPIs (a) Schematic of VPI measurements between DiI‐labelled NK‐CDVs and mixed (ICAM‐1/CD9) surfaces. ICAM‐1–mEGFP and mCherry–CD9 were co‐expressed from HEK cells and pulled down independently using two antibodies, namely, anti‐GFP and anti‐RFP. (b) Fluorescence images of surface‐tethered ICAM‐1 (*green*) and CD9 (*red*) either prepared alone (in “ICAM‐1 only” and “CD9 only”) or together by co‐expression (in “ICAM‐1/CD9”). DiI‐labelled NK‐CDVs interacting with the indicated surfaces are shown on right (*yellow*). Scale bars, 10 μm. (c) Fluorescence intensities measured from ICAM‐1 and CD9 surfaces (such as shown in (b)), with varying concentrations of input lysate. (d, e) VPIs of NK‐CDVs measured on ICAM‐1‐only surfaces (*green*), on CD9‐only surfaces (*red*), and on mixed surfaces with both ICAM‐1 and CD9 (*blue*) ICAM‐1‐only and CD9‐only data were fitted with density‐dependent cooperation models; mixed sample (ICAM‐1/CD9) data with a piecewise simple adsorption model. The upper axes were calculated from the intensity in the lower axes. (f) Comparison of measured (*solid*) versus expected VPIs (*dotted*) for mixed surfaces with ICAM‐1 and CD9. The expected curve was calculated as the sum of contributions from ICAM‐1‐only and CD9‐only samples (measured in (d) and (e) and plotted again as dotted curves in (f)). In all panels, error bars represent mean ± s.d. for *n* = 5−10 images, which are technical replicates acquired from distinct surface locations prepared with the same materials.

Recombinant CD9 protein was prepared by co‐expressing mCherry‐tagged CD9 (mCherry–CD9) with ICAM‐1–mEGFP in HEK cells. After cell lysis, both proteins were pulled down onto the glass surface that was covered with an equimolar mixture of two antibodies, namely anti‐GFP and anti‐RFP (Figure 4a). The surface attachment was purely random and independent so that the distances among the molecules would be determined by chance. Pull‐down from varying input concentrations yielded several samples that contained similar amounts of ICAM‐1 and CD9 (Figure 4b), as expected from their comparable concentrations in bulk. Additionally, to decompose the contributions from individual proteins and solely assess the synergy between ICAM‐1 and CD9, we also prepared surface‐tethered ICAM‐1 and CD9 alone, each in the same density ranges as in species in the mixed sample (Figure 4c). The isolated mCherry spots were subjected to photobleaching analysis (Figure S7A, B) similar to ICAM‐1–mEGFP, and again confirmed the single‐molecule nature of the fusion protein.

We then conducted VPI measurements for all three cases in parallel (Figure 4d,e). VPIs were strongly promoted on the surfaces with ICAM‐1 and CD9 mixtures (*blue* in Figure 4d,e) versus individual proteins in similar amounts (see also Figure 4b for image comparison, and Video S3 for real‐time images). This increase was saturated in the medium density range of ICAM‐1 (1−2 molecules/μm^2^), in which VPIs were possibly limited by vesicle diffusion toward the binding surface. Because of the fast increase in the low‐density regime, the measured VPIs were better explained by simple adsorption (followed by a plateau) than by a density‐dependent model with an increasing cooperativity (Supporting Information). More surprisingly, VPI levels on the mixed surface was much higher than the sum of contributions expected from individual species (Figure 4f). We suggest that this result can only be explained by the molecular‐level cooperation between ICAM‐1 and CD9, despite the restricted motions of surface‐tethered proteins.

### 2.9 Proximity of CD9 to ICAM‐1 enables cooperative VPIs

If the proximity between ICAM‐1 and CD9 molecules enables synergistic VPIs, their endogenous complexes would also promote vesicle binding in live cells. We therefore attempted to collect the putative ICAM‐1/CD9 complexes via single‐molecule co‐immunoprecipitation (co‐IP) (Jain et al., 2012; Lee et al., 2018) and analyse their vesicle‐binding potential using our method. We first prepared a surface with anti‐GFP to pull down ICAM‐1–mEGFP from the lysates of ICAM‐1/CD9 co‐expressing cells and checked whether mCherry–CD9 was pulled down together. However, we did not observe a specific increase in mCherry–CD9 signal above the background level, despite the single‐molecule sensitivity of our setup (Figure S7C, D; “Untreated”). The reverse pull‐down with anti‐RFP also yielded similar results,that is, no increase in GFP signal. Technically, these results confirmed that the cross‐reactivity of anti‐GFP and anti‐RFP, as well as the spectral bleed‐through between fluorescent channels, was minimal.

The lack of co‐IP between ICAM‐1 and CD9 indicates that their complexes were either not formed or dissociated during preparation. We therefore fixed the co‐expressing HEK cells with 2% paraformaldehyde prior to lysis so that the CD9 molecules in the proximity of ICAM‐1 can be crosslinked. In this way, a decent amount of ICAM‐1–CD9 complexes were obtained (Figure S7C, D; “Fixed”). A ratiometric analysis showed that 7% of ICAM‐1 was bound to CD9 (or a CD9/ICAM‐1 molar ratio of 1:14), and 11% of CD9 to ICAM‐1 in reverse. Importantly, the co‐pulled‐down fluorescent spots were not any brighter in fluorescence than the antibody‐pulled spots, which implies that the fixation procedure did not induce unnatural clustering of the overexpressed proteins.

**FIGURE 5:**
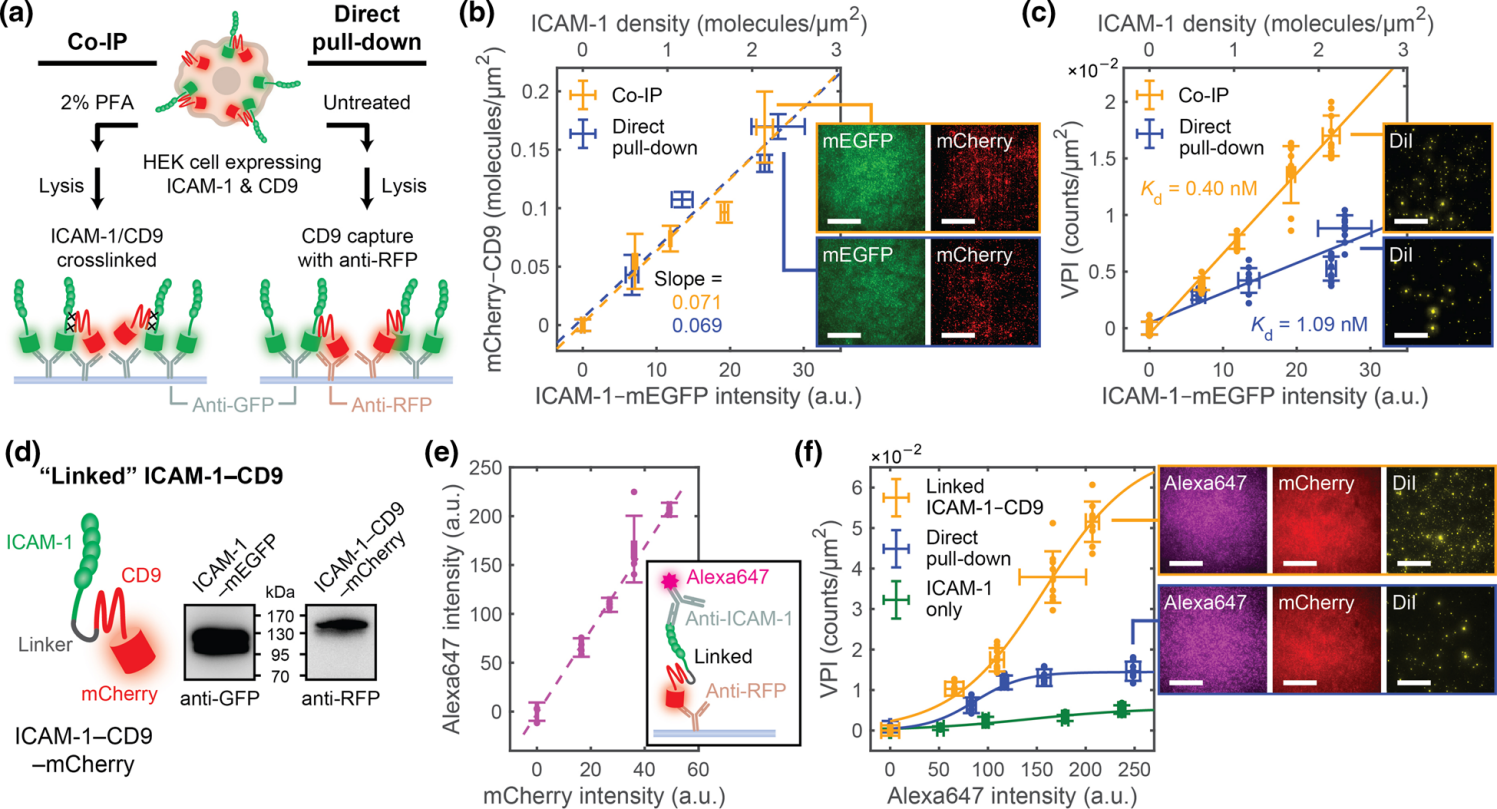
Proximity of CD9 to ICAM‐1 enables cooperative VPIs (a) Schematic of ICAM‐1/CD9 co‐immunoprecipitation (co‐IP) experiments after crosslinking. For co‐IP samples, HEK cells co‐expressing ICAM‐1–mEGFP and mCherry–CD9 were fixed with 2% paraformaldehyde (PFA) and ICAM‐1–mEGFP molecules were pulled down after lysis using anti‐GFP. For direct pull‐down, the co‐expressing cells were lysed without fixation and applied to a surface with both anti‐GFP and anti‐RFP, similarly to Figure 4a. (b) Fluorescence of ICAM‐1–mEGFP (intensity) and mCherry–CD9 (spot counts) measured from mixed surfaces prepared by co‐IP (*orange*) and direct pull‐down (*blue*). Dashed lines indicate linear fits. (c) VPIs measured between NK‐CDVs and ICAM‐1/CD9 mixed surfaces prepared by co‐IP and direct pull‐down. Data were fitted (*solid*) with simple adsorption models to obtain the equilibrium constants. In (b) and (c), the upper axis was calculated from the intensity in the lower axis. Insets, representative images from each sample that displayed similar amounts of surface proteins (scale bars, 20 μm). (d) Design of linked ICAM‐1/CD9 construct. Insets, western blot for the fluorescent protein tags in ICAM‐1–mEGFP and ICAM‐1–CD9–mCherry, verifying an increase in the overall length. (e) Detection of ICAM‐1 in linked ICAM‐1/CD9 construct by single‐molecule immunoblotting. The fluorescence from Alexa‐647‐conjugated ICAM‐1 antibody linearly increased with mCherry fluorescence. (f) VPIs measured between NK‐CDVs and linked ICAM‐1–CD9–mCherry (*orange*), mixed ICAM‐1–mEGFP and mCherry–CD9 via direct pull‐down (*blue*), and ICAM‐1–mEGFP only (*green*). For direct pull‐down sample, CD9 was prepared at a fixed, maximal density to maximize potential cooperation with ICAM‐1. Data were fitted (*solid*) with sigmoidal curves for guidance. Insets, representative images from each sample that displayed similar amounts of surface proteins (scale bars, 20 μm). In all panels, error bars represent mean ± s.d. for *n* = 5−10 images, which are technical replicates acquired from distinct surface locations prepared with the same materials.

We then asked whether the small pool of CD9 tightly bound to ICAM‐1 can elicit strong VPIs. Two sets of mixed surfaces with ICAM‐1 and CD9 were prepared by using different approaches (Figure 5a). The first method (“co‐IP”) used only anti‐GFP to pull down ICAM‐1 from the co‐expressing lysates, in which CD9 can be pulled down via co‐IP (same as the above paragraph). The second method (“direct pull‐down”) used a mixture of anti‐GFP and anti‐RFP to independently capture ICAM‐1 and CD9. This scheme is the same as that used in Figure 4a, except that the fraction of anti‐RFP was reduced to match the small portion of CD9 in the co‐IP samples (1:14 CD9/ICAM‐1). Thus, we indeed acquired two series of mixed samples that display nearly identical CD9/ICAM‐1 ratios across a wide density range (Figure 5b). Because the density of mCherry–CD9 was low in this experiment, CD9 spots were directly counted instead of conversion from intensity.

The complexes of ICAM‐1 and CD9 in the co‐IP samples showed a remarkable potency in VPI measurements (Figure 5c). The ICAM‐1 dependence of vesicle binding increased 2.7 times (slope change) relative to the untreated, direct pull‐down counterpart. VPIs in the direct pull‐down samples decreased from the earlier result (compare *blue* curves in Figures 4d and 5c), consistent with the reduced amount of CD9. Since the difference in VPIs between the co‐IP and direct pull‐down methods was observed for the same amounts of ICAM‐1 and CD9 (Figure 5b), the additional boost by co‐IP likely results from the proximity between ICAM‐1 and CD9 prescribed by crosslinking, in contrast to the random arrangement after direct pull‐down.

### 2.10 Linked ICAM‐1–CD9 enables strong VPIs

While the crosslinking approach can readily produce a tight complex of ICAM‐1 and CD9, it may also pull down other associated proteins, as well as disrupt the native activity of the proteins. We therefore prepared a fusion protein in which ICAM‐1 and CD9 are directly linked via a 6‐amino‐acid linker (Figure 5d) and tested it in our VPI assay. This ICAM‐1–CD9 fusion preserved the natural N‐to‐C directionality of both ICAM‐1 and CD9, and thus would be properly oriented for cooperation. It also carried mCherry on its C‐terminal for quantification and tethering, similarly to ICAM‐1‐ and CD9‐only constructs. The size of the expressed protein was checked by western blot and showed a small but distinct increase from ICAM‐1–mEGFP (Figure 5d, *inset*), indicating successful translation of the full‐length construct. In addition, the linkage between ICAM‐1 and mCherry was verified by single‐molecule immunoblotting. In this assay, the linked construct was first captured on glass surface by anti‐RFP (targeting mCherry) and then detected by Alexa647‐labelled anti‐ICAM‐1 (Figure 5e, *inset*). We observed that the resulting Alexa647 fluorescence increased linearly with mCherry fluorescence (Figure 5e), suggesting a stoichiometric relationship between ICAM‐1 and mCherry and also the integrity of ICAM‐1. Therefore, we concluded that the linked ICAM‐1–CD9 construct was prepared as designed.

When subjected to VPI imaging after pull‐down with anti‐RFP, the linked ICAM‐1–CD9 exhibited strong binding to NK‐CDVs, certainly stronger than ICAM‐1–mEGFP alone prepared at the same density (Figure 5f, *orange* vs. *green*). Since the two constructs were tethered by different antibodies, we exploited the ICAM‐1‐staining method (Figure 5e, *inset*) to normalize VPI counts to the amount of ICAM‐1 (and thus in Figure 5f, the VPI counts were plotted against Alexa647 intensity). We finally investigated whether the effect of ICAM‐1–CD9 linkage surpasses that of simple co‐attachment via direct pull‐down (Figure 5f). In order to test a wide range of mixing ratio (and of the resulting proximity) of ICAM‐1 and CD9 that may be encountered in physiological milieu, we gradually increased the amount of ICAM‐1 while maintaining CD9 at a maximal density to maximize potential cooperation from random proximity (Figure 5f, *blue*). Clearly, none of these conditions met the level of VPIs observed with the linked ICAM‐1–CD9. Collectively, these results suggest that the direct coupling of ICAM‐1 and CD9 might be a mechanism for the activation of ICAM‐1‐dependent vesicle adhesion.

### 2.11 Lipid membranes further support strong VPIs

**FIGURE 6:**
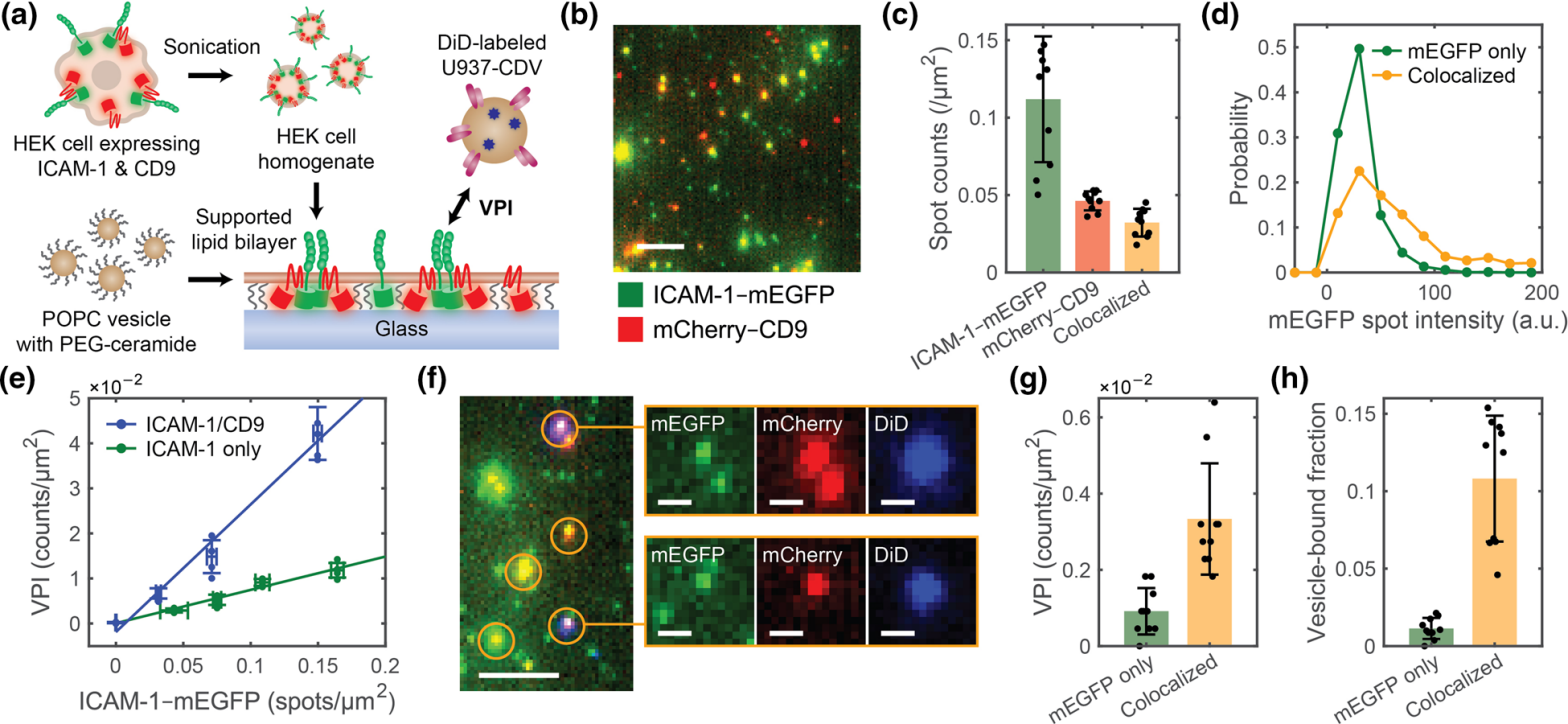
Lipid membranes further support strong VPIs (a) Schematic of SLB preparation followed by VPI measurements with membrane‐embedded proteins. (b) A representative fluorescence image of membrane‐embedded ICAM‐1–mEGFP (*green*) and mCherry–CD9 (*red*). Scale, 5 μm. (c) Counts of total ICAM‐1–mEGFP (*green*), total mCherry–CD9 (*red*), and their colocalized spots (*orange*). (d) Intensity distributions of mEGFP fluorescence for the mEGFP‐only (*green*) and colocalized spots (*orange*) detected in (c). *n* = 1744 (mEGFP only) and 706 (colocalized) spots. (e) VPI measured between U937‐CDVs and SLBs containing ICAM‐1 only (*green*) and mixed ICAM‐1 and CD9 (*blue*). Data were fitted (*solid*) with simple adsorption models to obtain the equilibrium constants. (f) Fluorescence images of membrane‐embedded ICAM‐1–mEGFP (*green*), mCherry–CD9 (*red*), and interacting DiD‐labelled U937‐CDVs (*blue*). Scale bars, 5 μm (*left*) and 1 μm (*right*). (g) Analysis of fluorescent spots enabling VPIs on SLBs. At the locations of DiD‐labelled CDVs, the numbers of mEGFP‐only and mEGFP/mCherry colocalized spots were counted. (h) Vesicle‐bound fraction of mEGFP‐only and mEGFP/mCherry colocalized spots. Values were calculated by dividing the numbers in (g) (spots interacting with vesicles) by the total numbers of the respective species. In all panels, error bars represent mean ± s.d. for *n* = 5−10 images, which are technical replicates acquired from distinct surface locations prepared with the same materials.

To complement the developed method that utilized surface‐tethered proteins without lipid membranes, we devised a scheme to examine the same VPIs on lipid bilayers (Figure 6a). To prepare supported lipid bilayers (SLBs) that contain target membrane proteins, HEK cells expressing ICAM‐1–mEGFP and mCherry–CD9 were disrupted by homogenization, and the resulting membrane homogenates were induced to form SLBs jointly with synthetic liposomes on a bare glass surface (Pace et al., 2015). The obtained SLBs were homogeneous and fluidic, as checked by the rapid diffusion of dye molecules (with diffusion coefficients in the range of 0.5−1.0 μm^2^/s) (Figure S8A–C).

Importantly, both ICAM‐1–mEGFP and mCherry–CD9 successfully partitioned into the SLBs (Figure 6b), the density of which can be controlled by adjusting the mixing ratio of membrane homogenates to synthetic liposomes (Figure S8D). We intentionally kept the density of proteins in SLBs low (up to 0.15 spots/μm^2^; Figure S8D) because we hoped to avoid excess incorporation of unwanted membrane proteins. Nevertheless, colocalization of ICAM‐1 and CD9 was remarkably high (Figure 6c), suggesting that such complexes would have assembled in live cells and remained intact, and further that they might serve as “hot spots” where several molecules can cooperate to capture vesicles. When ICAM‐1 and CD9 were prepared at similar densities, about 70% of the CD9 molecules colocalized with ICAM‐1. In contrast, ∼30% of ICAM‐1 was colocalized with CD9 because ICAM‐1 was expressed in excess. Among the ICAM‐1 population, the spots colocalized with CD9 tended to show brighter GFP signal than those without CD9 (Figure 6d), consistent with the role of CD9 in clustering ICAM‐1 (Franz et al., 2016).

Finally, we measured VPIs on SLBs that contained either ICAM‐1 only or both ICAM‐1 and CD9 (Figure 6e). We focused on examining U937‐CDVs to see whether their strong VPIs can be further enhanced by the presence of CD9 in native‐like membranes. Strikingly, we detected very strong interactions of U937‐CDVs with membrane‐embedded proteins, despite the very low density of proteins relative to surface‐tethering experiments. For example, VPIs on membranes were clearly distinguishable above background when ∼0.05 spots/μm^2^ ICAM‐1 was present (Figure 6e), but a similar level of VPIs was observed with ∼0.5 spots/μm^2^ surface‐tethered ICAM‐1 (Figure 1e). This represents an order‐of‐magnitude increase in vesicle‐binding potential in membrane environment (see Figure S8E for a direct comparison).

As with tethered proteins, the presence of CD9 distinctly promoted interactions between vesicles and ICAM‐1 (Figure 6e). To directly verify the participation of CD9 in this process, we examined the individual spots of fluorescent proteins and checked the colocalization of ICAM‐1, CD9, and DiD‐labelled vesicles (Figure 6f). Indeed, the interacting vesicles were mostly found on the spots that harbour both ICAM‐1 and CD9 (Figure 6g). The results were even more impressive when accounting for the large excess of ICAM‐1‐only spots over the colocalized spots (Figure 6c), which would have competed for vesicle binding (Figure 6h). This comparison suggests another order‐of‐magnitude increase—in addition to the membrane effect mentioned above—in the ICAM‐1‐mediated VPIs upon the participation of CD9. Together, these results collectively suggest that VPIs can be markedly strengthened when the transmembrane domains of membrane proteins are stably situated in lipid bilayers, and thereby enable formation of functional protein complexes.

## 3 DISCUSSION

In this work, we applied quantitative fluorescence microscopy to observe the functional interactions between single protein molecules and vesicles, especially investigating the effects of protein organization on PEG‐coated glass and on lipid bilayers. Although many studies have shown single‐vesicle imaging and analysis (e.g. using antibodies to capture vesicles and examine their content) (Colombo et al., 2021; Gebara et al., 2022; Han et al., 2021; He et al., 2019; Mutch et al., 2011; Zhou et al., 2020) and there exist other techniques to probe the target binding of vesicles (Ricklefs et al., 2018; Zhang et al., 2022), our approach is unique in that the molecular level cooperativity, as well as the thermodynamic and kinetic aspects of vesicle binding, can be quantitatively assessed. This information will be useful especially because EVs can not only be internalized upon target membrane binding but also directly stimulate intracellular signalling via cell‐surface receptors. The strength of VPI imaging is a direct consequence of wide‐field TIRF microscopy that allows monitoring of hundreds of vesicles in the field of view at high spatiotemporal resolution with single‐molecule sensitivity. Using this method, we discovered that molecular proximity between ICAM‐1 and CD9 enables effective capturing of vesicles, without the need to invoke a higher‐order organizing scheme for ligands and receptors. We also found distinct behaviours of NK‐ and U937‐CDVs in their binding to ICAM‐1, which demonstrated the utility of our approach. Finally, we showed that the method can be applied to natural EVs by employing NK‐cell‐derived EVs. Although we have not yet performed extensive tests with various EVs, such an extension is very plausible, given the physicochemical similarity between EV‐mimetic nano‐vesicles and EVs (Jang et al., 2013; Jo et al., 2014; Vázquez‐Ríos et al., 2019).

CD9 is traditionally believed to exert its effect through nanoscale clustering of ICAM‐1 (Barreiro et al., 2005, 2008; Franz et al., 2016), but we reported another mode of synergy between ICAM‐1 and CD9 (Figure 4), possibly involving multiple binding domains on LFA‐1. This synergy was even more prominent when we crosslinked ICAM‐1 and CD9 (Figure 5), which was quite surprising given the small population of CD9 relative to ICAM‐1 (7% or 1:14). We think that the closely interacting molecules might function as “hotspots” for vesicle binding. Consistent with this notion, locking ICAM‐1 and CD9 into a single complex strongly promoted VPIs. Moreover, VPIs on SLBs in which ICAM‐1 and CD9 were colocalized were extremely efficient, scoring the highest count per target protein (Figure 6). This trend occurred presumably because the lipid environment granted the membrane proteins more stability than surface tethering and facilitated their lateral registration. It must be emphasized that, while surface tethering with antibodies offers a simpler way to anchor protein molecules, reconstituting lipid bilayers would be crucial particularly if multiple transmembrane helices are involved, such as with tetraspanin proteins. Consistent with this argument, soluble ICAM‐1 without membrane incorporation shows a weak affinity toward LFA‐1 (Lee et al., 2010, p. 1; Meyer et al., 1995). CD9 has also been reported to regulate clustering of LFA‐1 in an inhibitory manner (Reyes et al., 2018, 2015), so a further investigation of the dual function of CD9 in more physiological conditions would provide additional insight.

The observation that NK‐ and U937‐CDVs exhibited VPIs with differential dependence on LFA‐1 (Figures 2 and 3) was quite intriguing. LFA‐1 normally shifts between three conformations: bent closed, extended closed, and extended open states, in the increasing order of affinity toward ICAM‐1 (Shimaoka et al., 2003). Mg^2+^ is one of the regulators of this change in LFA‐1 and subsequent cell adhesion and activation (Dransfield et al., 1992; Lötscher et al., 2022; Stewart et al., 1996), so the Mg^2+^‐dependent increase in VPIs with NK‐CDVs (Figure 3a) presumably relies on the same principle. Indeed, the fact that NK‐CDVs were brightly stained with an LFA‐1 antibody (HI111) that is specific to the closed, low‐affinity state of LFA‐1 (Ma et al., 2002) (Figure 3c) indicated that LFA‐1 activity in NK‐CDVs was largely suppressed before Mg^2+^ was added.

In contrast, U937‐CDVs sustained the activity from high‐affinity LFA‐1, which was insensitive to further regulation by Mg^2+^ (Figure 3b). One can consider the leukemic origin of U937 cells as the possible cause of activated LFA‐1, but mutations in integrins and their interactors that might enable the constitutive activity have not been reported (Barretina et al., 2012). Another possible explanation invokes the participation of effectors inside vesicles. For example, the cytoskeletal proteins talin and kindlin can both bind the β subunit of integrins and keep the cytoplasmic tails apart, thereby inducing a high‐affinity state (Kim et al., 2003; Moser et al., 2009). Recent evidence for the functional role of talin in exosomes (Soe et al., 2019) supports this idea. We speculate that the presence of a small number of effector molecules in vesicles might be sufficient to activate integrins because of high local concentrations in nano‐confinement (Shon & Cohen, 2012). Alternatively, nanoclusters of LFA‐1 assembled in U937 cells may have been enclosed in the CDVs and exhibited high avidity (or binding valency) toward ICAM‐1 surface (Bakker et al., 2012; Marwali et al., 2003; Shibagaki et al., 1999). To test different hypotheses, efforts to directly visualize the proteins that participate in VPIs are underway. Interrogating the mechanical aspects of integrin‐mediated VPIs with nano‐precision instruments may also yield useful insights (Yang et al., 2022).

Although the future extension of VPI imaging toward natural EVs and membranes would help tease out the physiological mechanisms of EV docking, it must be cautioned that the results presented here were mostly obtained with synthetic CDVs, so they may not be directly transferrable to EV research. In this vein, verifying the results with live cell adhesion and uptake assays in physiological conditions would be important. Unlike natural EVs that require significant efforts for collection and enrichment, CDVs can be obtained in a large amount by applying a relatively simple procedure (Jang et al., 2013), so they served as useful toy models to validate our assay. The peculiar traits of different types of CDVs that we discovered indicate that VPI imaging will prove useful to improve EV‐mimetic therapeutic vesicles (Jo et al., 2014; Vázquez‐Ríos et al., 2019). CDVs can be engineered to exhibit pharmaceutical activities, in which case the strength of their VPIs will correlate with their targeting ability. For example, CDVs can be generated from ligand‐activated or genetically modified cells to enrich specific components, and then subjected to VPI imaging to verify their potency. Thus, we foresee that our method can facilitate the screening of candidate vesicles in the developmental stage. Indeed, we discovered the remarkable potency of U937‐CDVs by using VPI measurements, which is hard to predict from the limited amount of LFA‐1. This result demonstrates the value of a facile semi‐functional test before application of laborious and costly assays that require cells. In this process, a novel drug target in the form of multimeric complexes (e.g. a cluster of ICAM‐1 (Taftaf et al., 2021)) might also be discovered.

## 4 CONCLUSION

In this study, we developed a technique that uses fluorescence microscopy to visualize vesicle–protein interactions at the single‐vesicle level. By observing the interactions between cell‐derived vesicles and ICAM‐1, we discovered that LFA‐1‐mediated VPIs strongly depend on vesicle types, close cooperation between ICAM‐1 and CD9, and the presence of lipid membranes. The method was also briefly shown to be applicable to natural EVs. We expect this technique can further help reveal the molecular mechanisms of cellular interaction for natural EVs and synthetic nano‐vesicles.

### 4.1 Materials and methods

#### 4.1.1 Preparation of vesicles

NK‐, U937‐, and UCMSC‐CDVs were prepared as previously described (Jang et al., 2013). Cells were first resuspended with phosphate‐buffered saline (PBS) at a concentration of 10^5^−10^6^ particles/mL, and then serially extruded through polycarbonate track‐etched hydrophilic membrane filters (Cytiva, Whatman) with pore sizes of 5, 1, and 0.4 μm for NK‐ and U937‐CDVs, and 10, 3, and 0.4 μm for UCMSC‐CDVs. Subsequently, CDV solutions were treated with DNase (Merck Millipore, Benzonase Nuclease HC) to remove DNA outside CDVs, and aggregates were removed by centrifugation at 3000 × *g* for 10 min. Then, CDVs were subjected to a tangential flow filtration system (Repligen, KrosFlo KR2i) to eliminate impurities such as free proteins or nucleic acids, followed by final filtration using a 0.45‐μm filter to remove large, non‐vesicular structures. The purified CDVs were stored in PBS at a concentration of ∼1×10^11^ particles/ml and kept at −80°C until use. For the preparation of NK‐cell‐derived EVs, human NK‐92 cells were grown and used as previously described (Choi et al., 2020). Briefly, NK‐92 cells were grown in α‐MEM supplemented with 2 mM L‐glutamine, 0.2 mM myo‐inositol, 0.02 mM folic acid, 0.1 mM 2‐mercaptoethanol, 20 ng/ml recombinant human IL‐2, 12.5% FBS, 12.5% horse serum and 1% penicillin/streptomycin. After 24 h of incubation in 10% EV‐depleted FBS, the cells were removed by centrifuging the solution once at 500 × *g* and twice at 2000 × *g*. The supernatant containing vesicles was then concentrated using tangential flow filtration system (Minimate TFF capsule) with a 100‐kDa membrane (Pall Corporation). The EVs were then purified by density‐gradient centrifugation (0.8 M and 2.0 M sucrose cushion and centrifugation at 100,000 × *g* for 2 h at 4°C) and further by iodixanol buoyant density gradient ultracentrifugation. The concentration and size distribution of CDV and EV samples were measured by NTA (Particle Metrix, Zetaview), DLS (Malvern, Zetasizer Nano ZS), and electron microscopy (negative‐stain TEM and cryo‐EM), along with total protein measurements to check vesicle purity (Table S1). When applicable, the protein content of vesicle samples was checked by SDS‐PAGE and western blot. Detailed methods and results for the characterization of vesicles are described in Supplementary Information. For fluorescent labelling of vesicles, vesicle solutions were prepared at 10^10^ particles/mL and reacted with 2 μg/mL DiI or DiD for 20 min at 37°C. The excess dye that remained as large aggregates was removed by filtering through a 0.2‐μm syringe filter (Advantec, 13CP045AS). The complete removal of excess dye was checked by measuring the fluorescence change after filtering, and the retrieval of labelled vesicles was checked by NTA.

#### 4.1.2 Preparation of proteins

For the construction of recombinant ICAM‐1–mEGFP and mCherry–CD9, cDNA fragments for ICAM‐1 and CD9 were cloned into CMV‐driven expression vectors containing fluorescent proteins. Linked ICAM‐1–CD9, CD11a–mEGFP, and CD18–mCherry were all prepared cloned similarly (for more details, see “Plasmid construction” in Supporting Information). For mammalian expression of recombinant proteins, the plasmids were transfected into HEK293 cells using polyethyleneimine (Sigma) for transient expression. Cells were grown at 37°C, 5% CO_2_, in Dulbecco's modified Eagle's medium (Sigma) supplemented with 10% foetal bovine serum (Welgene) and 1% penicillin‐streptomycin (Welgene) for 24 h and harvested by centrifuging at 500 × *g* for 10 min. The harvested cells were stored at −80°C until use. For preparing LFA‐1 proteoliposomes, recombinant LFA‐1 proteins (CD11a–mEGFP and CD18–mCherry) were expressed from Expi293F cells (Gibco) and purified with Ni‐NTA resin using a 6xHis‐tag on CD11a, and then reconstituted into synthetic liposomes (for more details, see “Preparation of liposomes with recombinant LFA‐1” in Supporting Information). For all fusion proteins, protein concentrations were measured with a fluorimeter (Shimadzu RF‐5301 PC).

#### 4.1.3 Construction of sample chambers

Sample slides for TIRF microscopy were prepared as previously described (Lee et al., 2018). Briefly, sample chambers for imaging were constructed from a glass coverslip and a glass slide bonded together using double‐sided tape. Glass surface was coated with polyethylene glycols (PEG) (Laysan Bio, mPEG‐SVA, MW 5,000), 3% of which was tagged with biotin (Laysan Bio, BIO‐PEG‐SVA, MW 5,000) to tether NeutrAvidin (Thermo Fisher, 31000).

#### 4.1.4 VPI imaging with surface‐tethered proteins

For surface tethering of proteins, cell pellets expressing ICAM‐1–mEGFP and/or mCherry–CD9 were lysed with 1% Triton X‐100 in PBS (Biosesang, pH 7.2). For ICAM‐1/CD9 co‐IP experiments, cells were crosslinked with 2% PFA for 10 min before lysis. After removing cell debris by centrifugation, the concentrations of fluorescent proteins were measured with a fluorometer. For VPI imaging, solutions of NeutrAvidin, biotin‐conjugated antibodies (anti‐GFP: Abcam, ab6658; anti‐RFP: Abcam, ab34771), and the cell lysates containing fluorescent proteins were sequentially injected into a sample chamber, with 5−10 min of incubation for each step followed by washing with PBS. When necessary, fluorescence of surface‐tethered proteins was imaged and quantified before VPI measurements. Then, fluorescently labelled CDVs were introduced into the chamber, allowed to react for 10 min, and washed with PBS to remove unbound vesicles. For VPI blocking experiments, CDVs were first treated with the respective antibodies (anti‐CD11a (BioLegend, 301202), anti‐CD18 (Abcam, ab8220) (5 μg/ml), or anti‐RFP (Abcam, ab28664) as a negative control), similarly to immunoblotting assay below, and then subjected to VPI measurements.

#### 4.1.5 VPI imaging on SLBs

For preparing SLBs that contain the membrane proteins expressed from HEK cells, HEK cell homogenates and synthetic PEGylated liposomes were separately prepared and mixed to induce the formation of SLBs (Pace et al., 2018). For synthetic liposomes, 2 mg of 1‐palmitoyl‐2‐oleoyl‐sn‐glycero‐3‐phosphocholine (POPC; Avanti, 850457C) dissolved in chloroform was mixed with 7.4 μg of PEG5000‐ceramide (Avanti, 880280P). When the fluorescence imaging of membrane was necessary, 0.002−2 nmol of DiD was optionally included, but not for general VPI imaging. Lipids were completely dried under vacuum and resuspended with 1 mL PBS, and the resulting solution was extruded through 100‐nm pores (Avanti, 610023). For HEK cell homogenates, ∼4 × 10^7^ cells expressing ICAM‐1–mEGFP and/or mCherry–CD9 were harvested and disrupted by homogenization (DH.WHG02016). The crude mixture was subjected to multiple cycles of centrifugation to remove large debris (1000 × *g* for 10 min, and then 9000 × *g* for 5 min twice, collecting the supernatant in between). The supernatant was centrifuged again at 100,000 × *g* for 30 min twice to pellet membrane‐derived vesicles. The pellets were resuspended in PBS and mixed with 0.4 mg/mL of synthetic liposomes, typically at a ratio from 5:95 to 20:80 (*v*:*v*), and then sonicated for 15 min to induce the fusion of vesicles. The solution of hybrid vesicles, after mixing with an equal volume of 2× PBS, was loaded onto a clean glass surface to generate SLBs. After 15 min of incubation, vesicle solution was removed and the surface was washed with PBS. Finally, CDV samples were loaded onto the bilayers for VPI measurements.

#### 4.1.6 Single‐vesicle immunoblotting assay

For the detection of LFA‐1, unlabelled CDVs (1×10^10^ particles/ml) were incubated with anti‐CD11a (BioLegend, 301202) or anti‐CD18 (Abcam, ab8220) antibodies (5 μg/ml) for 1 h at 4°C. Vesicles were collected by centrifugation at 18,000 × *g* for 1 h and resuspended in PBS, and the supernatant containing unbound free antibodies was discarded. The centrifugation step followed by resuspension was repeated once for further purification. For imaging, antibody‐bound CDVs were captured on the PEG‐coated surface of a sample chamber via biotinylated secondary antibody (Jackson ImmunoResearch, 715‐065‐150) and NeutrAvidin. Cy3‐conjugated secondary antibody (Jackson ImmunoResearch, 715‐165‐150) directed to anti‐CD11a and anti‐CD18 was then introduced for single‐vesicle staining.

#### 4.1.7 TIRF microscopy and image analysis

Sample slides were imaged on a home‐built TIRF microscope using an inverted microscope (Olympus IX73) and a 60× oil‐immersion lens for both illumination and imaging. Wavelengths of lasers used for illumination (Cobolt) were: 488 nm for mEGFP, 532 nm for mCherry, DiI, and Cy3, and 633 nm for DiD. Images were acquired by an sCMOS camera (Teledyne Photometrics, Prime BSI Express) typically with 100‐ms resolution and ∼100‐μm field of view. For quantification of fluorescence, initial 10 frames of movies were averaged and either the number of fluorescent spots or the total intensity over the entire area was measured using custom MATLAB codes. VPI counts were extracted by detecting diffraction‐limited fluorescent spots (i.e. local maxima of the image) whose intensity exceeded a custom threshold (Figure S3A, B). For the conversion of fluorescence intensity to surface density, calibration curves for mEGFP or mCherry were obtained from the images of low‐density samples. The calibration was performed individually for each dataset to ensure accurate conversion. Before any colocalization analysis, optical crosstalk between fluorescent channels was carefully corrected for by using individual species of surface‐tethered fluorescent proteins.

## AUTHOR CONTRIBUTIONS

M.J.S. conceived the study. M.C., S.H.J., J.J., and M.J.S. designed experiments and analysed data. M.C., S.H.J., and J.H.P. conducted vesicle labelling and VPI measurements. J.J. performed western blotting and immunoblotting of vesicles. C.S.L. and J.J. performed electron microscopy, J.‐O. L. supervised the process, and J.J., S.H.J., and M.J.S. analysed EM images. Y.B. prepared CDVs and S.‐S.P. and S.W.O. supervised the process. S.B. prepared NK‐EVs and Y.S.G. supervised the process. M.J.S. developed VPI models. M.J.S. wrote the manuscript with support from M.C., S.H.J., and J.J. and input from all authors. S.W.O. provided important scientific comments. M.J.S. supervised all aspects of the project.

## CONFLICT OF INTEREST STATEMENT

M.C., Y.B., S.W.O., and M.J.S. filed a patent on VPI imaging described in this study.

## Supporting information

Supporting Information

Supporting Information

Supporting Information

Supporting Information
