# Supplementary material for: Quantitative imaging of vesicle–protein interactions reveals close cooperation among proteins
Source: J Extracell Vesicles. 2023 Apr 25;12(5):12322. doi: 10.1002/jev2.12322 (PMC10130417; doi:10.1002/jev2.12322)
Supplement: Supplementary file 1 — Supporting Information [file JEV2-12-12322-s002.pdf]

Supporting Information for:

Quantitative imaging of vesicle–protein interactions reveals close cooperation among proteins

Cha *et al.*

This PDF file includes:

Supplementary Methods
Modeling of vesicle–protein interactions
Supplementary Figures S1–S8
Supplementary Table S1
Supplementary Video Legends
Supplementary References

Supplementary Methods

Plasmid construction

The ICAM-1–mEGFP plasmid was constructed by removing the 15F11-HA gene from the pCMV-15F11-HA-mEGFP (Addgene, 129590; kindly provided by Dr. Jong-Bong Lee at POSTECH) and cloning the human ICAM-1-coding ORF (Sino Biological, HG10346-UT) into the vector. The mCherry–CD9 plasmid (Addgene, 55013) was purchased and used as received. For the cloning of ICAM-1–CD9–mCherry linked construct, the ORF fragments for ICAM1, CD9, and mCherry were separately amplified and fused together by overlap extension PCR. To avoid steric hindrance, a short linker $(GGS)_2$ was placed between ICAM-1 and CD9. This insert fragment was then ligated into a linearized vector that was produced by digesting mCherry–CD9 plasmid with NheI and BamHI. The plasmids for recombinant LFA-1 proteins were prepared similarly (HiFi DNA Assembly) by cloning cDNA fragments into the plasmids synthesized above. Specifically, to generate CD11a–mEGFP from the ICAM-1–mEGFP plasmid, the ICAM-1 sequence was replaced with human CD11a ORF (Sino Biological, HG10812-M). Similarly, to generate CD18–mCherry from the ICAM-1–mEGFP plasmid, both the ICAM-1 and mEGFP sequences were replaced with human CD18 (Sino Biological, HG10970-M) and mCherry ORFs, respectively. For both fusion proteins, a flexible linker consisting of $(GGGGS)_5$ was introduced between the LFA-1 subunit and the fluorescent protein. The CD11a–mEGFP construct contained an additional 6xHis-tag in the middle of the linker for affinity purification. In all cases, joining of dsDNA fragments were performed by either NEBuilder HiFi DNA Assembly (New England Biolabs, E2621S) or Gibson Assembly (New England Biolabs, E2611S), without notable difference.

Single-molecule photobleaching analysis

To verify that the observed fluorescent spots consist of single molecules of the prepared recombinant protein (ICAM-1–mEGFP and mCherry–CD9), protein-coated areas (prepared at a low density to avoid overlapping of spots) were illuminated until most of the observed spots were photobleached (Figures S1C and S7A) (Ulbrich & Isacoff, 2007; Kim et al., 2021). The fluorescence from each spot displayed a stepwise decrease in intensity, characteristic of single-molecule photobleaching events. To estimate the number of molecules in each spot, hidden Markov modeling was performed on each trace while varying the expected number of states from 1 to 5, and the corresponding Bayesian Information Criteria (BIC) for the best-fit models were calculated:

$$\mathrm{BIC} = k\ln(N) - 2\ln(\hat{L}), \tag{1}$$

where k is the number of output parameters from the model, N is the number of data points, and $\hat{L}$ is the log-likelihood function of the model. In this way, the number of states that can model the fluorescence signal well enough without too much overfitting can be determined as the point at which the BIC curve changes abruptly (Choi et al., 2019; Lee, 2009). Then, the photobleaching step size was measured from the observed bleaching events (Figures S1D and S7B), which followed the lognormal distribution as expected (Mutch et al., 2007). Finally, the numbers of fluorophores (i.e. fluorescent protein molecules) per puncta were estimated by dividing the initial fluorescence before photobleaching by the mean step size (exp(1.72) = 5.6 for mEGFP and exp(1.47) = 4.3 for mCherry). Analysis of multiple images showed that the majority of the spots (66% of ICAM-1−mEGFP and 74% of mCherry−CD9) contained only single molecules (Figures S1D and S7B).

Immunoprecipitation of ICAM-1–mEGFP

To check for the presence of ICAM-1-associated proteins, ICAM-1–mEGFP was immunoprecipitated from the expressing HEK cell lysate (containing 3 μg of ICAM-1–mEGFP) by using 1 mg of magnetic beads (Thermo Fisher, 65305) coated with 5 μg of anti-GFP (Abcam, ab6658). After 2 h of incubation at room temperature (RT) with gentle shaking, the beads were washed twice with 0.1 % BSA in PBS. Proteins were then eluted by incubation with 0.1 M glycine (pH 2.5) for 10 min with gentle shaking and neutralized with 1 M Tris (pH 8.0). The eluent was subsequently mixed with SDS lysis buffer and boiled at 95 °C for 10 min. After separation by SDS-PAGE on a non-reducing 10% gel, protein bands were visualized by staining with Coomassie brilliant blue R-250.

Particle analysis of vesicle samples

For nanoparticle tracking analysis (NTA), both CDV and EV samples were diluted with filtered PBS to ~10^9 particles/ml and illuminated with 405-nm laser in the NTA equipment (NanoSight, LM10-HS). About 30 measurements were performed to analyze each vesicle sample. All the vesicle samples we used were sized similarly with median diameters in the range of 120–160 nm (Table S1). For a complementary measurement of particle dispersion, DLS was used to measure the polydispersity index of vesicle samples. The obtained values were around or below 0.3 for all samples (Table S1) and similar to the values reported for EVs and their mimetics (Vázquez-Ríos et al., 2019), which suggests fairly homogeneous vesicles that are well dispersed without aggregation. For transmission electron microscopy (TEM), CDV samples (10 μl) were loaded on a formvar-coated grid (Electron Microscopy Science) for 30 min at RT, and then negatively stained with 10 μl of 2% uranyl acetate solution for 10 s. After that, samples were dried at RT for 24 h and

imaged (JEOL JEM-1011). For TEM image analysis, we employed a recently developed deep-learning software (Gómez-de-Mariscal et al., 2019) to segment vesicle images, and the resulting labeled images were used to calculate equivalent diameter and roundness (= $4\pi(\text{area})/(\text{perimeter})^2$; 1 for a perfect circle) of the identified particles (Figure S2D). The resulting size distributions overall agreed with NTA results (Figure S2E), except for the systematic omission of smaller particles in NTA analysis (Bachurski et al., 2019). The roundness distributions for all three CDV samples strongly peaked at 1 (Figure S2E), again confirming the spherical shapes (roundness greater than 1 is associated with pixilation artifact of small objects). For cryo-electron microscopy (cryo-EM), 3.5 μl of EV samples (10^{11} particles/ml) were applied to glow-discharged 300-mesh Quantifoil R 1.2/1.3 holy carbon grids (Quantifoil Micro Tools). After 10 s of incubation, excess samples were removed by blotting for 5 s and plunge-frozen in liquid ethane using Vitrobot Mark IV (Thermo Fisher Scientific). The vitrified EV images were acquired at 200 kV with a Talos Glacios microscope (Thermo Fisher Scientific) equipped with a Falcon IV direct electron detector (Thermo Fisher Scientific). The images were recorded at 73,000× magnification (pixel size 1.41 Å) with a total dose of 30 e/Å^2 and varying defocus ranges from −2.0 to −3.0 μm using EPU software (Thermo Fisher Scientific). To examine the morphology of the CDVs, we manually inspected the obtained vesicle images and categorized the particle shape into either unilamellar, multilamellar, or non-spherical (tubular) (Table S1). The majority of all three CDVs possessed a spherical, unilamellar structure, validating the use of CDVs as a simple model of membrane-bound particles. Finally, we measured the protein content of the CDV samples, which are summarized in Table S1. The resulting particle-to-protein ratio, which is an indicative of vesicle purity, agreed with the previously reported values for cell-derived vesicles (Jang et al., 2013). The slightly higher

protein content in CDVs than natural EVs is known and thought to result from nonselective incorporation of proteins into vesicles as opposed to the biogenesis of EVs (Jo et al., 2014).

Chemical analysis of vesicle samples

For sodium dodecyl sulfate-polyacrylamide gel electrophoresis (SDS-PAGE) analysis of proteins in CDV samples, NK-, U937-, and UCMSC-CDVs, together with the cell pellets of their origin, were lysed with 1% Triton X-100 (Sigma) and their total protein concentrations were measured (Bio-Rad, 5000112). Then, equal amounts (4 μg) of protein were loaded onto 8% polyacrylamide gel for separation and stained with Coomassie brilliant blue R-250. For western blotting, equal concentrations of CDV samples (2×10^9 particles/ml) were lysed with SDS (Sigma). The samples were loaded onto 6–10% (for CD11a, CD18) and 12% (for CD63) polyacrylamide gels for separation and transferred to polyvinylidene difluoride (PVDF) membranes. The transferred membranes were blocked using 5% skim milk in tris-buffered saline (TBS) for 1 h at RT. Then, the membranes were incubated overnight with primary antibodies against CD11a (BioLegend, 301202), CD18 (Abcam, ab176540), and CD63 (BioLegend, 353017) at 4 °C. After that the membranes were washed with Tween-20 in TBS (TBST) and incubated with secondary antibodies (Abcam, ab205718 and ab205719) for 1 h at RT, then washed with TBST again. The bands were visualized by chemiluminescence reagent (Cytiva, RPN2232). For the western blot of NK-EVs, additional antibodies for CD81 (Santa Cruz sc-166029), CD63 (Santa Cruz sc-5275), LFA-1 (CD11a; BD 610826), calnexin (BD 610523), actin (Santa Cruz sc-47778), and H2B (Cell Signaling 8135S) were used to check for EV markers and controls (Figure S5C).

Preparation of liposomes with recombinant LFA-1

For the expression of CD11a–mEGFP and CD18–mCherry, 120 ml of Expi293F cells (Gibco A14528) were grown (8 % CO_2 with shaking at 120 rpm) to the density of 3×10^6 cells/ml. The cells were co-transfected with 60 μg of cDNAs coding for CD11a–6xHis–mEGFP and CD18–mCherry using Expi293F transfection kit (Gibco A14524). After 3 d, cells were collected by centrifuging the suspension at 300×g for 5 min, resuspended in Dulbecco's PBS (Sigma) for washing, and then harvested again by centrifugation at 500×g for 5 min. The collected cells were snap-frozen and kept at −80 °C until use. For purification, LFA-1-expressing cells were lysed with 12 ml of ice-cold lysis buffer (20 mM HEPES (pH 7.4) with 300 mM NaCl, protease and phosphatase inhibitor cocktails (Sigma P8340, P5726, and P0044; 1% each), and 2 % Triton-X100) and further homogenized (DH.WHG02016). The crude lysate was centrifuged at 15,000×g to remove cell debris and then passed through 2 ml of nickel-charged resin bed twice (4 ml of homogeneous QIAGEN Ni-NTA Agarose). The resins were washed with 20 ml of wash buffer (50 mM HEPES (pH 7.4) with 300 mM NaCl, 20 mM imidazole, and 0.2 % Triton X-100), then with 20 ml of the wash buffer with 1 % octyl β-D-glucopyranoside (OG; Glycon) instead of Triton X-100, and then eluted with 5 ml of 50 mM HEPES (pH 7.4) with 300 mM NaCl, 400 mM imidazole, 10 % glycerol, and 1 % of OG. The protein solution was concentrated with 100-kDa-cutoff centrifugal filter (Amicon UFC900324), replacing the buffer to 50 mM HEPES (pH 7.4) with 150 mM NaCl, 10% glycerol, and 1 % OG. The final protein concentrations were measured by fluorimeter using the fluorescent protein tags. The presence of CD11a and CD18 was verified by western blotting (Figure S6A) and single-molecule fluorescence analysis (Figure S6B–D), and the function of LFA-1 was checked by Mn^{2+}-dependent increase in the active-form population (Figure S6G,H). To prepare proteoliposomes, purified LFA-1 subunits were mixed with synthetic lipids

(99.9 mol% of 1-palmitoyl-2-oleoyl-glycero-3-phosphocholine (POPC) (Avanti 850457P) and 0.1 mol% of DiD and OG) so that final solution contained 0.375 μM of LFA-1 (with respect to CD11a–mEGFP), 3 mM lipids, and 2 % of OG, and incubated for 30 min at 4 °C. The mixture was then diluted three times by adding 50 mM HEPES (pH 7.4, 150 mM NaCl) and incubated with activated SM-2 resins at 4 °C for 6 h with agitation for detergent removal. The prepared proteoliposomes were kept at 4 °C and used within 1 day of preparation. For the nonspecific capturing of liposomes with cholesterol (Figure 2K), a short duplex DNA (IDT) that is tagged with cholesterol and biotin was prepared from two oligos (He et al., 2017):

5′-biotin-TTTTTTGCAGAAATAAGGCACGAGCTTT-cholesterol-3′

5′-cholesterol-TTTGCCGTCGTGCCTTATTTCTGC-3′

and was tethered to the PEG-coated glass surface instead of anti-GFP and ICAM-1−mEGFP.

Fluorescence recovery after photobleaching (FRAP) analysis

To verify the fluidity of supported lipid bilayers (SLBs), FRAP experiments were performed. The fractional recovery of fluorescence intensity (normalized to the initial fluorescence) over time, $f(t)$, in a uniform, circular region of interest with a radius ω was modeled by the following expression (Soumpasis, 1983):

$$f(t) = e^{-\frac{2\tau_D}{t}}\left[I_0\left(\frac{2\tau_D}{t}\right) + I_1\left(\frac{2\tau_D}{t}\right)\right], \qquad (2)$$

where $\tau_D = \omega^2/4D_t$ is the characteristic diffusion time with a diffusion coefficient D_t, and I_0 and I_1 denote the modified Bessel functions. In our experiments, ω ~ 9 μm was used, but the precise value of ω did not greatly affect the obtained diffusion coefficients.

Modeling of vesicle–protein interactions

Simple adsorption model

At the simplest level, vesicle–protein interactions (VPIs) can be described by the Langmuir adsorption isotherm, similarly to its application in protein-ligand binding. In this case, target protein molecules on glass surface serve as randomly distributed adsorbents for vesicles. If the vesicle docking is a single-step process with uniform affinity, the equilibrium VPI counts (N_V) is expected to follow:

$$N_V = N_V^{max} \frac{c_V}{K_\mathrm{d} + c_V} = \frac{d_P c_V}{K_\mathrm{d} + c_V} A \,, \tag{3}$$

where N_V^{max} is the maximum capacity for VPI, c_V the vesicle concentration, and K_d the associated equilibrium constant. The second equality in Eq. (2) simply substitutes N_V^{max} with the functional amount of target protein (such as ICAM-1) in the field of view, $N_P = d_P A$ (d_P: protein density; A: area), assuming monovalent VPIs. In some cases, this expression was sufficient to describe the measured VPIs. For example, the linear increase of U937-CDVs interacting with ICAM-1 (Figure 1E) suggests that K_d remains constant across the range of ICAM-1 density.

Density-dependent cooperation among proteins

When surface-tethered ICAM-1 is prepared at high densities, cooperation among two or more ICAM-1 molecules to capture single vesicles is conceivable. We thus developed a kinetic model for multivalent VPIs (Figure S4):

a) Each vesicle is first captured by a single protein molecule with an on-rate constant k_on.
b) *Single-tether sites*: If the captured vesicle cannot find additional protein molecules within its reach, it unbinds with an off-rate constant $k_\mathrm{off,1}$ (Figure S4A).

c) *Double-tether sites*: Alternatively, if another molecule of surface-tethered protein locks onto the membrane of the captured vesicle (not necessarily via the same mechanism as the first tether), the double-tethered vesicle unbinds slowly with an effective off-rate constant $k_{\mathrm{off},2}$ (Figure S4B).

For the case c), we expect that the formation of the second tether would be practically instantaneous because the effective concentrations of vesicles and proteins in the local vicinity are very high. Consequently, VPIs on double-tether sites can be described with an effective equilibrium constant of $K_{\mathrm{d},2} = k_{\mathrm{off},2}/k_{\mathrm{on}}$, which is smaller compared with the single-tether equilibrium with $K_{\mathrm{d},1} = k_{\mathrm{off},1}/k_{\mathrm{on}}$. This model then becomes equivalent to a two-site Langmuir adsorption model accounting for the different types of sites with differing affinities:

$$N_V = \left[\frac{d_1 c_V}{K_{\mathrm{d},1} + c_V} + \frac{d_2 c_V}{K_{\mathrm{d},2} + c_V}\right] A\,, \tag{4}$$

where d_1 and d_2 denote the densities of potential single- and double-tether sites in the area A, respectively. In principle, more than two tethers per vesicle can be formed that will further reduce the effective K_{d}, but this small population is practically insignificant in the density regime we investigated (see below) and ignored in Eq. (3). Note that the proportion of d_2 versus d_1 is expected to increase with total density, $d_P = d_1 + 2d_2$, but is also related to vesicle size and tether geometry. By considering the dimensions of participating molecules (ICAM-1: 18.7-nm-long rod (Staunton et al., 1990); mEGFP: 4-nm-tall barrel; immunoglobulin G (IgG) antibody: 14.5 nm × 8.5 nm Y-shape (Silverton et al., 1977)), we estimated that the maximum separation of two ICAM-1 molecules that can bind to a single 150-nm (median) vesicle is ~150 nm (Figure S4C). Then, the number of double-tether sites can be deduced from the distribution of nearest-neighbor distance, r_{NN}, for randomly distributed points on surface:

$$f(r_{\mathrm{NN}}; d_P) = 2\pi d_P r_{\mathrm{NN}} \exp(-\pi d_P (r_{\mathrm{NN}})^2) \,. \tag{5}$$

Representative distributions of r_{NN} for the typical values of d are plotted in Figure S4D. These examples show that indeed a decent fraction of ICAM-1 molecules may cooperate in binding single vesicles. By integrating Eq. (4) from 0 to r_{max} (the maximum separation, 150 nm), one can calculate the expected number of sites that can form double tethers (Figure S4E):

$$\begin{aligned} \Pr(r_{\mathrm{NN}} \leq r_{max}) &= \int_0^{r_{max}} 2\pi d_P r_{\mathrm{NN}} \exp(-\pi d_P (r_{\mathrm{NN}})^2) \, dr_{\mathrm{NN}} \\ &= 1 - \exp(-\pi d_P (r_{max})^2) \,, \end{aligned} \tag{6}$$

and therefore:

$$d_2(d_P, r_{max}) = \frac{1}{2} d_P [1 - \exp(-\pi d_P (r_{max})^2)] \,, \tag{7}$$

$$d_1(d_P, r_{max}) = d_P \exp(-\pi d_P (r_{max})^2) \,. \tag{8}$$

For triple tethers and so on, one can consider a generalized expression for the distribution of the *N*th nearest neighbor distance, $r_{\mathrm{NN},N}$:

$$f\left(r_{\mathrm{NN},N}; d_P\right) = \frac{2}{(N-1)!} (\pi d_P)^N \left(r_{\mathrm{NN},N}\right)^{2N-1} \exp\left(-\pi d_P \left(r_{\mathrm{NN},N}\right)^2\right) , \tag{9}$$

but these fractions are relatively minor in the density regime we analyzed (see Figure S4E, *yellow* for the population with the second-nearest-neighbor distance, $\Pr\left(r_{\mathrm{NN},2} \leq r_{max}\right)$). Additionally, the effects from these populations may not be readily distinguishable because even the double-tether sites appeared to be so sticky that the off-rate was extremely slow (Figure S3B). Finally, plugging in Eqs. (6) and (7) into Eq. (3), we obtain the model equation for VPI counts, $N_V\left(d_P; K_{\mathrm{d},1}, K_{\mathrm{d},2}\right)$, which we termed the "density-dependent cooperation" model. Calculations of representative curves are given in Figure S4F using the known values of r_{max} = 150 nm and c_V = 3.3 pM (the measured counts are typically normalized by the area *A* so the exact value of *A* is unnecessary),

with their comparison to simple adsorption models with single equilibrium constants, *i.e.* Eq. (2) (Figure S4G). Fitting the measured values of N_V and d_P with this model effectively captured the super-linear increase in VPI signals in the medium density regime (d_P ~ 1–3 molecules/μm^2) and returned reasonable values of equilibrium constants such as shown in Figure 1E.

Feasibility of multi-tether VPI

Molecular cooperation for single-vesicle binding can be argued as follows:

a) Assuming uniform distribution, 40 molecules/μm^2 (the maximum density in Figure 1C) gives 0.025 μm^2 per molecule, which corresponds to 160 nm of intermolecular spacing. According to Figure S4C, this separation would be just enough to form multiple tethers on some large single vesicles.
b) The real distribution of ICAM-1 molecules of course cannot be perfectly uniform, so this randomness brings some ICAM-1 molecules closer to each other than 160 nm.
c) In fact, the nearest-neighbor distance almost always benefits from the randomness, because it is very unlikely that all the surrounding molecules are located farther than the uniform case. It can be estimated from Eq. (4) that, for 40 molecules/μm^2, the nearest-neighbor distance is shorter than 160 nm for 96% of the surface-tethered molecules. In particular, the curves in Figure S4D show that, even at under 40 molecules/μm^2, decent fractions of ICAM-1 molecules will be endowed with their nearest neighbor within 150 nm, the maximum separation that allows multiple binding.
d) Finally, when one molecule grabs onto a vesicle, the effective vesicle concentration for the neighbors hugely increases, and therefore multiple tethers would be readily formed if geometrically feasible.

VPI counts limited by mass transport of vesicles

If VPIs between a certain pair of vesicles and proteins consume too many vesicles in the vicinity of the protein surface, vesicle binding would be limited by the mass transport of vesicles toward the surface, similarly to the known behavior in surface plasmon resonance experiments (Schuck

& Zhao, 2010). Since we used a stationary reservoir of vesicles during the incubation period, the limited diffusion of vesicles would lead to the formation of a depletion zone near the surface, which will limit the maximum count of bound vesicles. Examples of this kind are shown in Figure 4D and E for the mixed surfaces (*blue*). The onset of such effect can be rationalized by the increased surface-binding of vesicles, especially in the context of low vesicle concentration that we used (~3 pM). Without further considering vesicle diffusion kinetics, these observations can be described by a simple adsorption curve that asymptotes to the maximum level. The data were therefore fitted to a piecewise linear equation, and the slope of the beginning phase was used to extract the equilibrium constant: $(\mathrm{slope}) = c_V/(K_\mathrm{d} + c_V)$.

Cooperativity among vesicles

The above descriptions of VPIs do not explain the dependence of VPIs on vesicle concentration shown in Figure 1F. We think this is because of the cooperative nature of VPIs in more concentrated solutions of vesicles. Although we could not conduct experiments at higher vesicle concentrations due to the sample limit, the super-linear increase in Figure 1F likely represents the initial rise of a sigmoidal adsorption curve (also known as S-shaped or Type V isotherm) (Rabe et al., 2011) that results from cooperative binding. Several models to describe such a cooperativity exist, but applying them to VPIs would be presumptuous at this point because the mechanistic basis of cooperativity in vesicle binding is still under investigation. Instead, we adopted a Hill coefficient, n, to assess the degree of cooperativity:

$$N_V = N_V^{max} \frac{(c_V)^n}{K_\mathrm{d} + (c_V)^n}. \qquad (10)$$

For example, in Figure 1F, the measured VPI counts were fitted with Eq. (9) to obtain the Hill coefficients of 2.2 and 1.2 for U937- and NK-CDVs, respectively.

Supplementary Figures

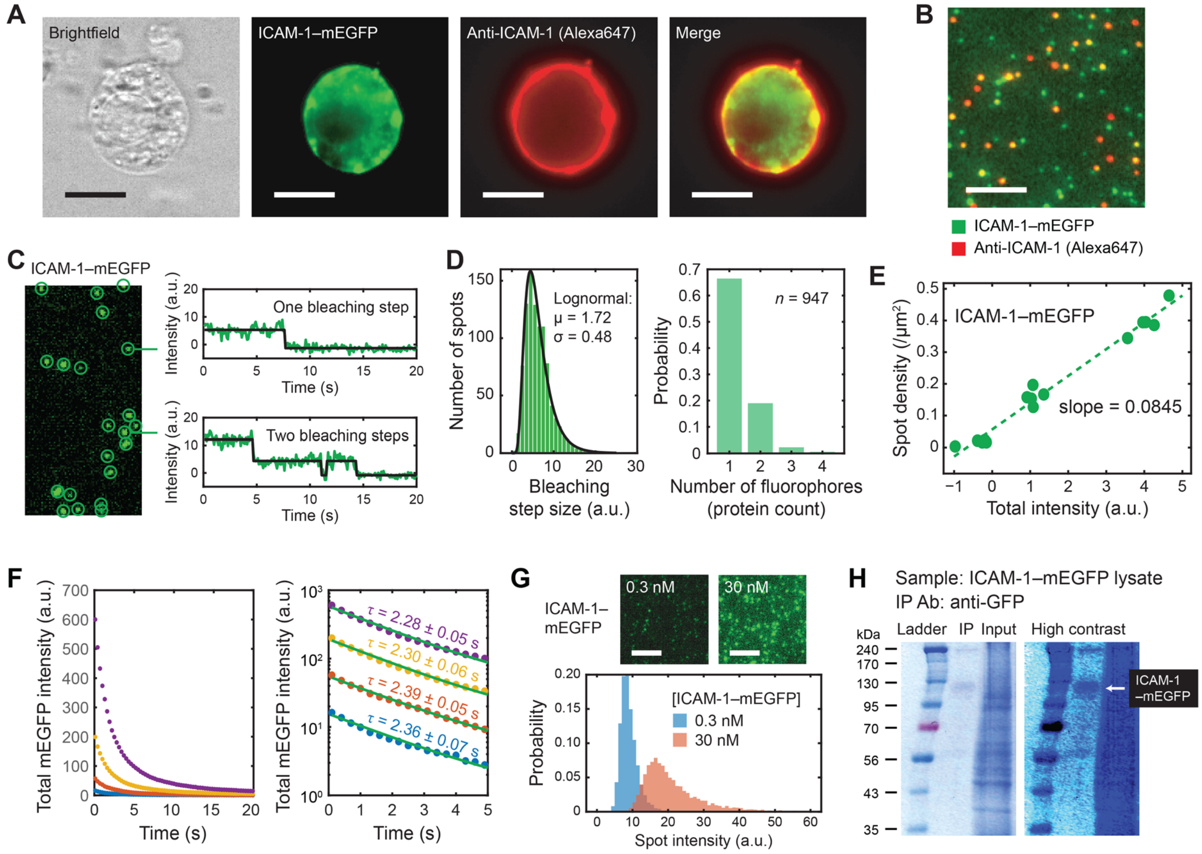

Figure S1. Verification of ICAM-1–mEGFP

(A) Micrographs of a representative HEK293 cell transfected with ICAM-1–mEGFP (*green*) and stained with Alexa647-conjugated ICAM-1 antibody (BioLegend 322718; 5 μg/ml) (*red*). Scale, 10 μm. (B) A representative fluorescence image of surface-tethered ICAM-1–mEGFP (*green*) stained with Alexa647-conjugated ICAM-1 antibody (*red*). Scale, 5 μm. Detection by the labeled antibody was not perfect because we used a low concentration of the antibody (1 μg/ml) to minimize nonspecific binding. (C) Photobleaching of ICAM-1–mEGFP spots. A representative image is shown with sample photobleaching traces (*green*) and the corresponding hidden Markov model (*black*) (see Supplementary Methods, "Single-molecule photobleaching analysis"). (D) Distribution of photobleaching step size and the estimated number of fluorophores per spot obtained from n = 947 spots. (E) Calibration of surface-tethered ICAM-1–mEGFP fluorescence

using low-density preparations. The obtained linear relationship was applied to Figs. 1C (blue dashed line) and 1E (top *x*-axis) in the main text. (F) Photobleaching of ICAM-1–mEGFP fluorescence prepared at different densities. The fluorescence decay was fitted with single-exponential models (solid green on right) and the resulting decay rate was universal across the prepared densities, indicating the absence of self-quenching behavior in dense samples. (G) Fluorescence images and the corresponding spot intensity distribution of surface tethered ICAM-1–mEGFP prepared at the indicated concentrations. For the 30 nM sample, the surface density of NeutrAvidin was lowered to resolve single spots of ICAM-1–mEGFP. Scale, 5 μm. Histograms were plotted for n = 5,192 and 8,166 molecules for 0.3 and 30 nM, respectively. (H) Proteins immunoprecipitated from ICAM-1–mEGFP-expressing HEK-cell lysate with anti-GFP-coated magnetic beads. A non-reducing 10% polyacrylamide gel with SDS was used to separate proteins and visualized with Coomassie brilliant blue R-250.

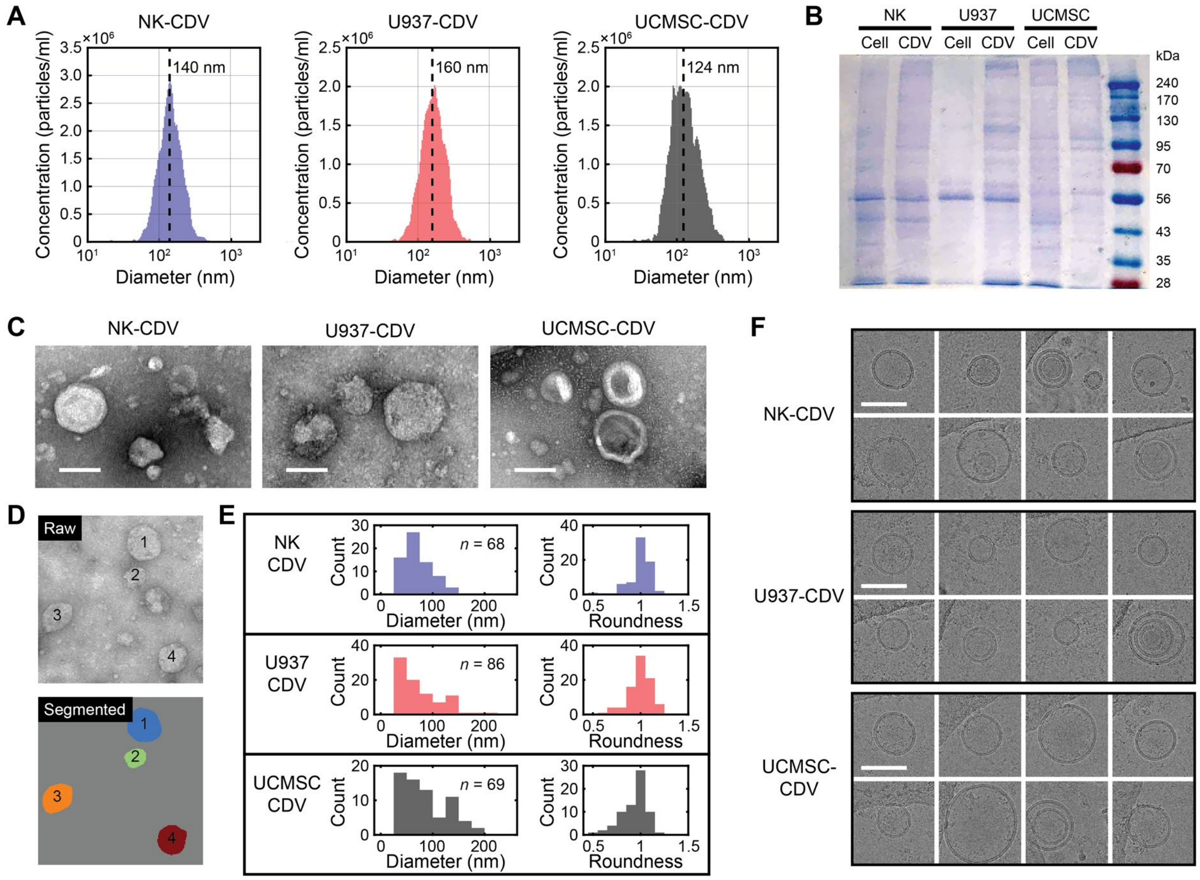

Figure S2. Characterization of CDVs

(A) NTA analysis of NK-, U937-, and UCMSC-CDVs. Dashed line, median diameter. (B) SDS-PAGE analysis of CDVs and their parental cells. For each sample, 4 µg of protein were loaded into each lane. 8% polyacrylamide gel was stained with Coomassie brilliant blue R-250 for visualization. (C) Representative negative-stain TEM images of CDVs. Scale, 100 nm. (D, E) Analysis of TEM images. A deep-learning-based image segmentation software (Gómez-de-Mariscal et al., 2019) was employed for automatic identification of vesicles (D), and then the labeled images were used to calculate equivalent diameter and roundness ($= 4\pi(\text{area})/(\text{perimeter})^2$) of the identified particles (E). (F) Representative cryo-EM images of CDVs. Scale, 100 nm. Results of particle analysis is summarized in Table S1.

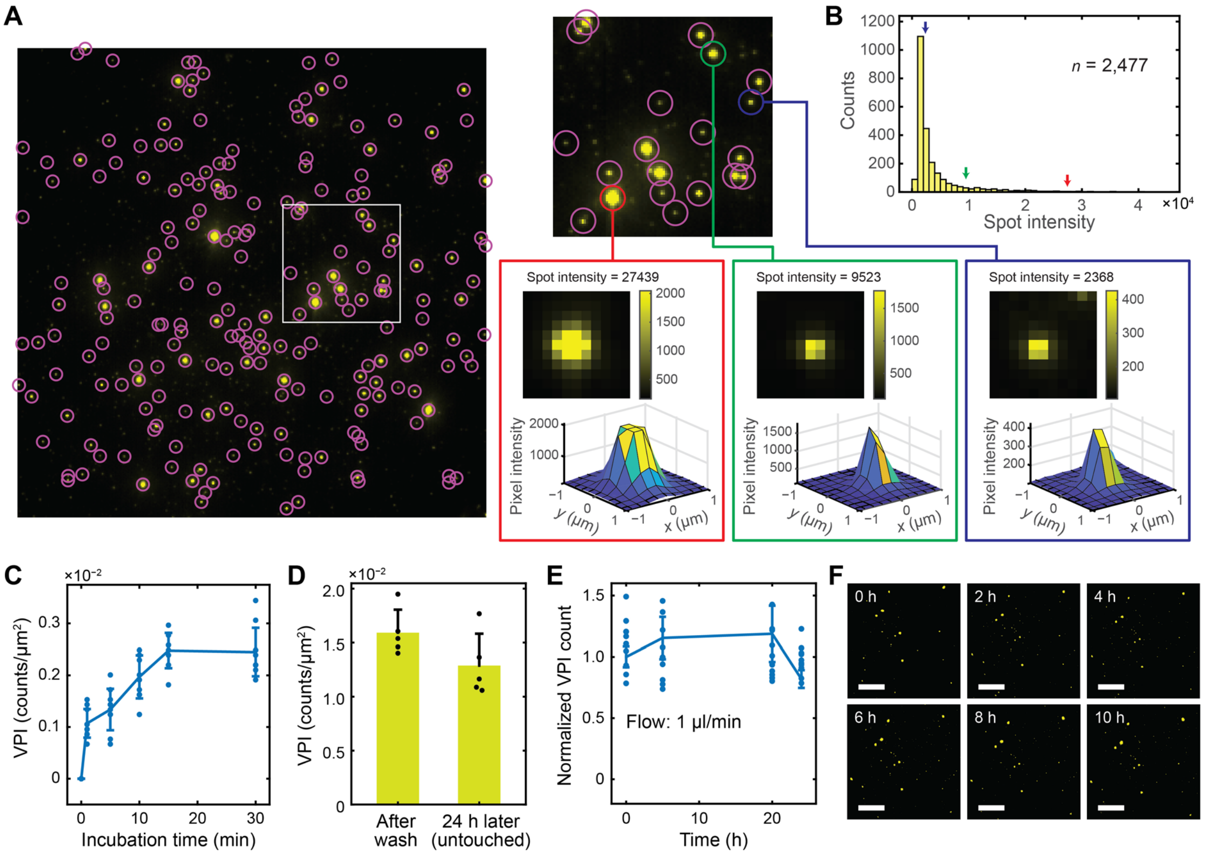

Figure S3. VPI analysis

(A) Detection of VPI. The image on left is the same as the one in Figure 1D (for 2.4 nM ICAM-1) in the main text. Magenta circles represent the programmatically detected vesicle spots. The region marked by a white box is expanded on right, and the spot images with varying levels of intensity are shown below. (B) Distribution of spot intensity obtained from 10 images, such as shown in (A). The colored arrows indicate the locations of the selected spots in (A). (C) VPI increase during the association phase of NK-CDV binding to surface-tethered ICAM-1–mEGFP. (D) Stability of VPI was checked after 24 h of incubation in PBS without perturbation. (E, F) VPI stability in the presence of a gentle flow (1 µl/min). The sample chamber (~7 µl volume) was continuously flushed with PBS for 24 h and the VPI counts were monitored during the wash. In (F), most of the vesicles in the selected region were checked to remain intact, except for slight displacement and photobleaching (scale bar, 10 µm). In all panels, error bars represent mean ± s.d. for n = 5–10 images, which are technical replicates acquired from distinct surface locations prepared with the same materials.

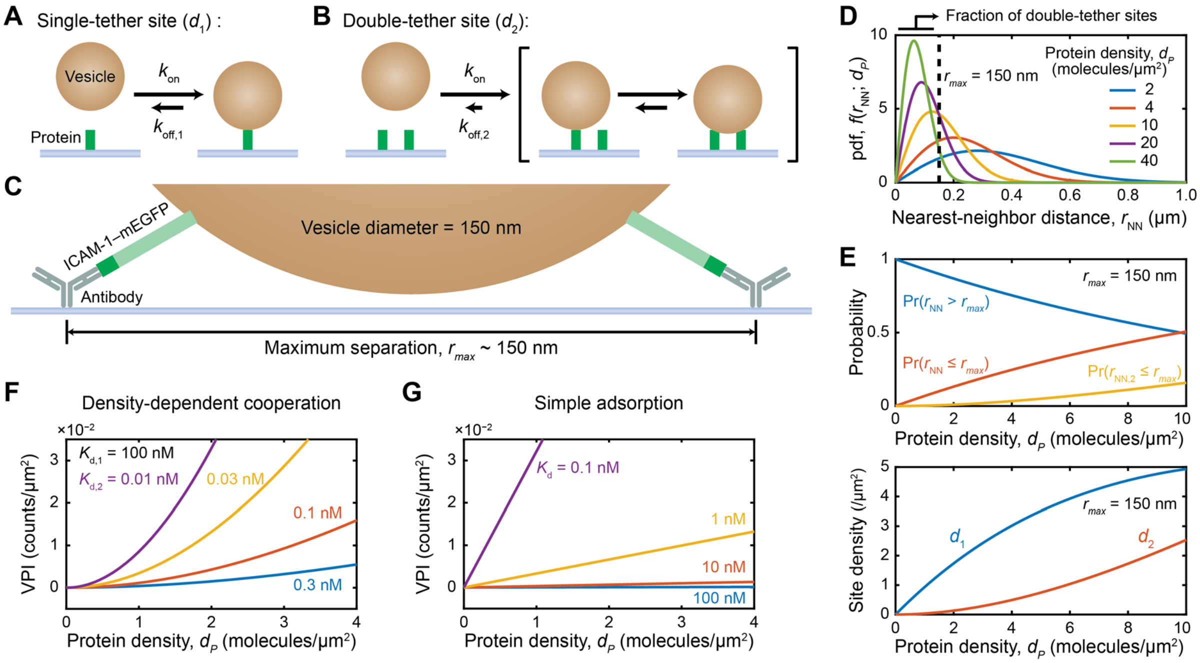

Figure S4. Modeling of VPIs

(A, B) Cartoons for VPI involving a single (A) and double tethers (B). See "Modeling of VPIs" for the detailed descriptions of the model. (C) Geometric estimation for the maximum separation of two ICAM-1 molecules that can form double tethers on a single vesicle. (D) Probability density functions (pdf) of nearest-neighbor distance, $f(r_{\mathrm{NN}}; d_P)$, for the indicated values of surface protein density (d_P). Calculations are based on Eq. (4). Dashed line indicates r_{max} = 150 nm, the maximum separation shown in (C). (E) Probabilities of sites that cannot (*blue*) and can form double tethers (*red*) calculated from Eq. (5) (*top*), with the corresponding expected numbers of single- (d_1) and double-tether sites (d_2), respectively (*bottom*). Yellow curve on top represents the probability for the second-nearest-neighbor distance calculated by integrating Eq. (8) from 0 to r_{max} = 150 nm. (F, G) VPI counts expected from density-dependent cooperation models (Eq. (3)) and from simple adsorption models (Eq. (2)). For both (F) and (G), the following values were used for calculations: r_{max} = 150 nm, c_V = 3.3 pM.

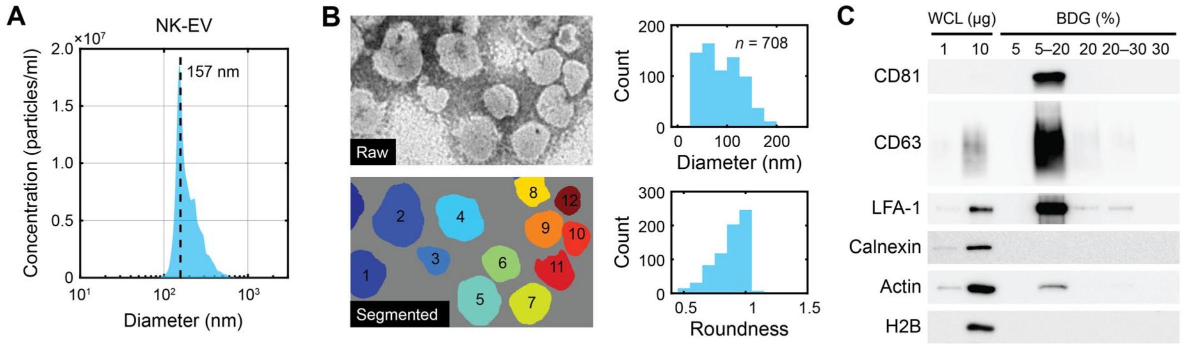

Figure S5. Characterization of NK-EVs

(A) NTA analysis of NK-EVs. Dashed line, median diameter. (B) TEM imaging and analysis of NK-EV sample. The deep-learning analysis to obtain size and roundness distribution was the same as in CDV analysis. (C) Western blot analysis of protein content in NK-EVs.

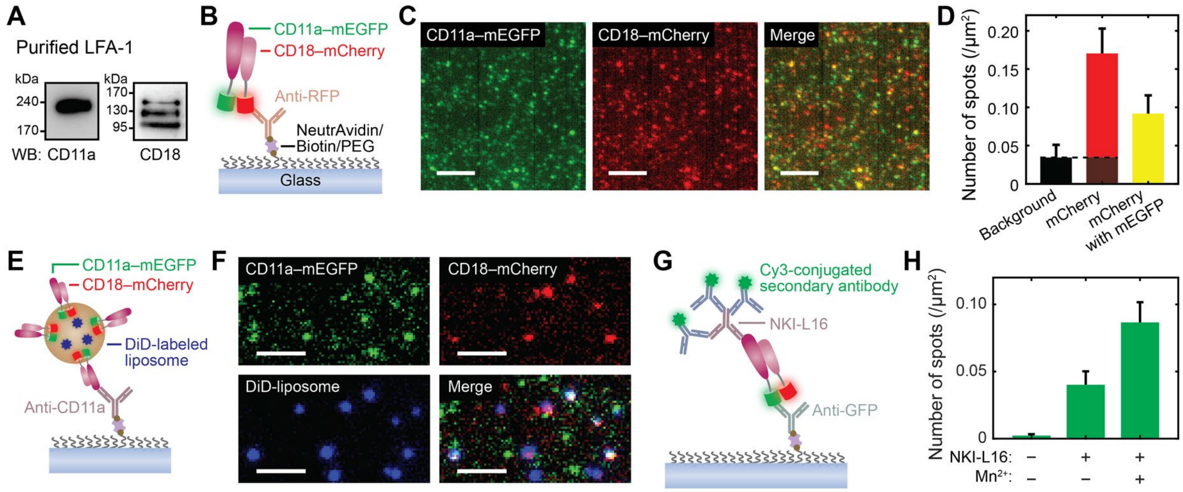

Figure S6. Verification of recombinant LFA-1 and its reconstitution in liposomes

(A) Western blot of purified LFA-1. Detection of CD11a and CD18 was performed the same way as with CDVs in Figure S2A. (B) Single-molecule pull-down of recombinant LFA-1 with antibody to mCherry (anti-RFP). (C) Representative images of CD11a–mEGFP and CD18–mCherry spots. Scale, 10 µm. (D) Colocalization of CD18–mCherry spots with CD11a–mEGFP signal. Correcting for the background count in the mCherry channel (*black*), which naturally does not contribute to colocalization, ~68% of CD18–mCherry spots were observed to colocalize with CD11a–mEGFP. (E, F) Three-color colocalization of CD11a–mEGFP, CD18–mCherry, and DiD-labeled liposomes. After capturing LFA-1-reconstituted proteoliposomes via CD11a, successful incorporation of LFA-1 subunits in liposomes was verified by the presence of mixed-colored puncta in the merged image (white spots if all three components are present). Scale, 5 µm. (G, H) Schematic and results of single-molecule immunolabeling of recombinant LFA-1 with activation-specific CD11a antibody (NKI-L16). The native conformational changes of LFA-1 expected from the addition of 5 mM Mn^{2+} was checked by the increased detection with NKI-L16.

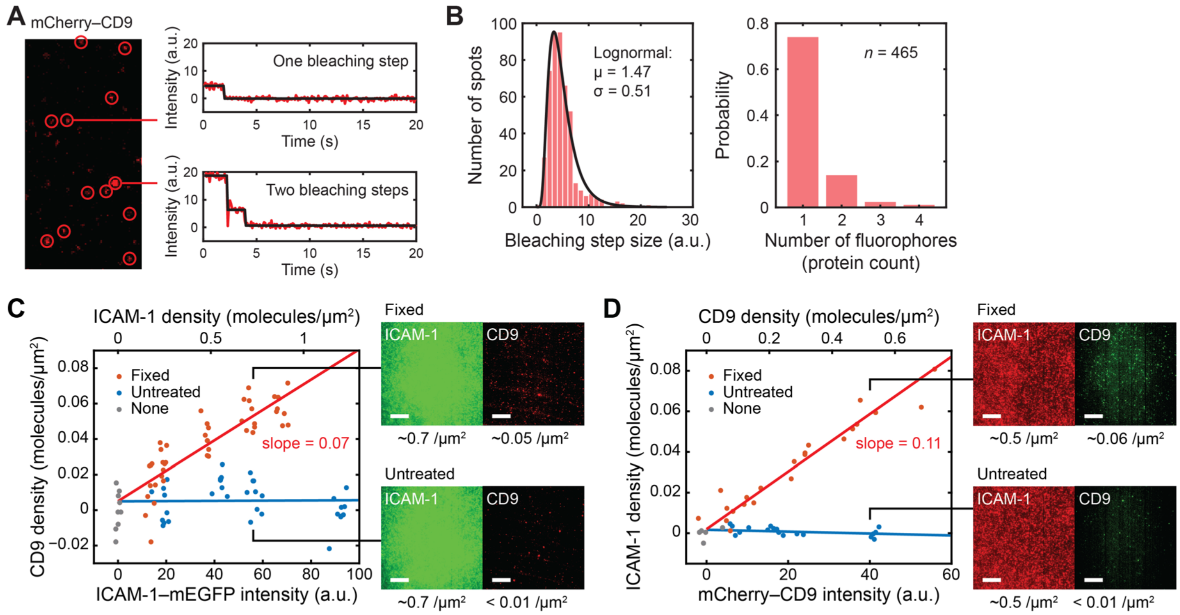

Figure S7. Single-molecule analysis of mCherry–CD9

(A) Photobleaching of mCherry–CD9 spots. A representative image is shown with sample photobleaching traces (*red*) and the corresponding hidden Markov model (*black*) (see Supplementary Methods, "Single-molecule photobleaching analysis"). (B) Distribution of photobleaching step size and the estimated number of fluorophores per spot obtained from $n = 465$ spots. (C, D) Single-molecule co-immunoprecipitation of ICAM-1 and CD9. Counts of CD–mCherry spots pulled down with ICAM-1–mEGFP (C), and those of ICAM-1–mEGFP spots pulled down with CD–mCherry are shown. For both (C) and (D), solid lines represent linear fits for fixed and untreated samples with the annotated values of slope. Insets, representative images from the indicated data that displayed similar amounts of pulled-down proteins. Scale, 10 μm.

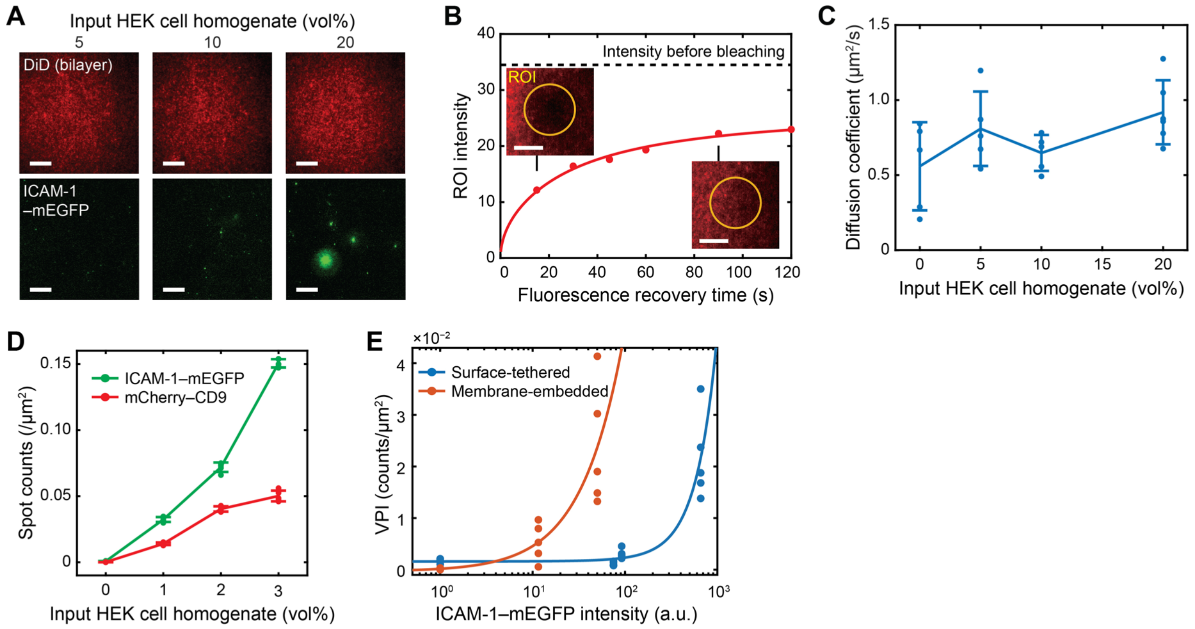

Figure S8. Verification of supported lipid bilayers

(A) Fluorescence images of DiD-labeled SLB (*red*) with membrane-embedded ICAM-1–mEGFP (*green*). (B) FRAP measurements for the analysis of the membrane fluidity in SLBs. The measured intensity during the recovery (*dots*) were fitted with a model (*solid*) described in Supplementary Methods. Insets, representative images of a photobleached region after 15 and 90 s of recovery. (C) Diffusion coefficients of DiD molecules in SLBs obtained from FRAP experiments as a function of input HEK cell homogenate amount. (D) Counts of ICAM-1–mEGFP (*green*) and mCherry–CD9 (*red*) spots observed in SLBs as a function of input HEK cell homogenate amount. (E) VPIs measured for U937-CDVs with increasing amounts of ICAM-1–mEGFP in surface-tethered (*blue*) and membrane-embedded (*red*) conditions. For the data in (C)–(E), multiple images are technical replicates acquired from distinct surface locations prepared with the same materials. In all panels, error bars represent mean ± s.d. for n = 5–10 images, which are technical replicates acquired from distinct surface locations prepared with the same materials.

Supplementary Table

Table S1. Properties of CDVs and NK-EV

	NK-CDV	U937-CDV	UCMSC-CDV	NK-EV
**Stock concentration (particles/ml)**	1.3×10^{11}	1.3×10^{11}	5.1×10^{11}	3.3×10^{11}
**Median diameter (from NTA)**	139.9 nm	160.1 nm	123.7 nm	157 nm
**PDI* (DLS)**	0.225	0.279	0.312	0.297
**Protein concentration**	238 µg/ml	168 µg/ml	254 µg/ml	50 µg/ml
**Particles/µg protein**	5.5×10^{8}	7.7×10^{8}	2.0×10^{9}	6.6×10^{9}
**Morphology** ** **(cryo-EM)**	(*n* = 53)	(*n* = 61)	(*n* = 48)	
Unilamellar	72%	70%	81%	
Multi-lamellar	19%	18%	8%	
Tubular	9%	11%	10%	

* PDI: Polydispersity index; ** Vesicle morphology after vitrification was manually inspected for the indicated numbers of vesicle images from cryo-EM.

Supplementary Video Legends

Video S1. Real-time images of fluorescently labeled vesicles

Fluorescence images of DiI-labeled NK-CDVs (*yellow*) freely diffusing over bare glass surface without target proteins. Images were acquired from a TIRF microscope at 30-ms time resolution.

Video S2. Sequence of VPI measurements

Demonstration of overall VPI imaging with TIRF microscopy.

Video S3. Real-time images of VPI measurements with TIRF microscopy

Fluorescence images of DiI-labeled U937-CDVs (*yellow*) interacting with surface-tethered ICAM-1–mEGFP (*green*) and mCherry–CD9 (*red*). This data was used to generate Figure 4B. Note that the images were not taken simultaneously from the same region but obtained from the same surface in a single sample chamber.

Supplementary References

Bachurski, D., Schuldner, M., Nguyen, P.-H., Malz, A., Reiners, K. S., Grenzi, P. C., Babatz, F., Schauss, A. C., Hansen, H. P., Hallek, M., & Pogge von Strandmann, E. (2019). Extracellular vesicle measurements with nanoparticle tracking analysis – An accuracy and repeatability comparison between NanoSight NS300 and ZetaView. *Journal of Extracellular Vesicles*, *8*(1), 1596016. https://doi.org/10.1080/20013078.2019.1596016

Choi, H.-K., Min, D., Kang, H., Shon, M. J., Rah, S.-H., Kim, H. C., Jeong, H., Choi, H.-J., Bowie, J. U., & Yoon, T.-Y. (2019). Watching helical membrane proteins fold reveals a common N-to-C-terminal folding pathway. *Science*, *366*(6469), 1150–1156. https://doi.org/10.1126/science.aaw8208

Gómez-de-Mariscal, E., Maška, M., Kotrbová, A., Pospíchalová, V., Matula, P., & Muñoz-Barrutia, A. (2019). Deep-Learning-Based Segmentation of Small Extracellular Vesicles in Transmission Electron Microscopy Images. *Scientific Reports*, *9*(1). https://doi.org/10.1038/s41598-019-49431-3

He, F., Liu, H., Guo, X., Yin, B.-C., & Ye, B.-C. (2017). Direct Exosome Quantification via Bivalent-Cholesterol-Labeled DNA Anchor for Signal Amplification. *Analytical Chemistry*, *89*(23), 12968–12975. https://doi.org/10.1021/acs.analchem.7b03919

Jang, S. C., Kim, O. Y., Yoon, C. M., Choi, D.-S., Roh, T.-Y., Park, J., Nilsson, J., Lötvall, J., Kim, Y.-K., & Gho, Y. S. (2013). Bioinspired Exosome-Mimetic Nanovesicles for Targeted Delivery of Chemotherapeutics to Malignant Tumors. *ACS Nano*, *7*(9), 7698–7710. https://doi.org/10.1021/nn402232g

Jo, W., Kim, J., Yoon, J., Jeong, D., Cho, S., Jeong, H., Yoon, Y. J., Kim, S. C., Gho, Y. S., & Park, J. (2014). Large-scale generation of cell-derived nanovesicles. *Nanoscale*, *6*(20), 12056–12064. https://doi.org/10.1039/C4NR02391A

Kim, C., Shon, M. J., Kim, S. H., Eun, G. S., Ryu, J.-K., Hyeon, C., Jahn, R., & Yoon, T.-Y. (2021). Extreme parsimony in ATP consumption by 20S complexes in the global disassembly of single SNARE complexes. *Nature Communications*, *12*(1), 3206. https://doi.org/10.1038/s41467-021-23530-0

Lee, T.-H. (2009). Extracting Kinetics Information from Single-Molecule Fluorescence Resonance Energy Transfer Data Using Hidden Markov Models. *The Journal of Physical Chemistry B*, *113*(33), 11535–11542. https://doi.org/10.1021/jp903831z

Mutch, S. A., Fujimoto, B. S., Kuyper, C. L., Kuo, J. S., Bajjalieh, S. M., & Chiu, D. T. (2007). Deconvolving Single-Molecule Intensity Distributions for Quantitative Microscopy Measurements. *Biophysical Journal*, *92*(8), 2926–2943. https://doi.org/10.1529/biophysj.106.101428

Rabe, M., Verdes, D., & Seeger, S. (2011). Understanding protein adsorption phenomena at solid surfaces. *Advances in Colloid and Interface Science*, *162*(1), 87–106. https://doi.org/10.1016/j.cis.2010.12.007

Schuck, P., & Zhao, H. (2010). The Role of Mass Transport Limitation and Surface Heterogeneity in the Biophysical Characterization of Macromolecular Binding Processes by SPR Biosensing. In N. J. Mol & M. J. E. Fischer (Eds.), *Surface Plasmon Resonance: Methods and Protocols* (pp. 15–54). Humana Press. https://doi.org/10.1007/978-1-60761-670-2_2

Silverton, E. W., Navia, M. A., & Davies, D. R. (1977). Three-dimensional structure of an intact human immunoglobulin. *Proceedings of the National Academy of Sciences*, *74*(11), 5140–5144. https://doi.org/10.1073/pnas.74.11.5140

Soumpasis, D. M. (1983). Theoretical analysis of fluorescence photobleaching recovery experiments. *Biophysical Journal*, *41*(1), 95–97. https://doi.org/10.1016/S0006-3495(83)84410-5

Staunton, D. E., Dustin, M. L., Erickson, H. P., & Springer, T. A. (1990). The arrangement of the immunoglobulin-like domains of ICAM-1 and the binding sites for LFA-1 and rhinovirus. *Cell*, *61*(2), 243–254. https://doi.org/10.1016/0092-8674(90)90805-O

Ulbrich, M. H., & Isacoff, E. Y. (2007). Subunit counting in membrane-bound proteins. *Nature Methods*, *4*(4). https://doi.org/10.1038/nmeth1024

Vázquez-Ríos, A. J., Molina-Crespo, Á., Bouzo, B. L., López-López, R., Moreno-Bueno, G., & de la Fuente, M. (2019). Exosome-mimetic nanoplatforms for targeted cancer drug delivery. *Journal of Nanobiotechnology*, *17*(1), 85. https://doi.org/10.1186/s12951-019-0517-8
